# Prediction and mechanistic analysis of drug-induced liver injury (DILI) based on chemical structure

**DOI:** 10.1186/s13062-020-00285-0

**Published:** 2021-01-18

**Authors:** Anika Liu, Moritz Walter, Peter Wright, Aleksandra Bartosik, Daniela Dolciami, Abdurrahman Elbasir, Hongbin Yang, Andreas Bender

**Affiliations:** 1 Department of Chemistry, Centre for Molecular Informatics, University of Cambridge, Lensfield Road, Cambridge, CB2 1EW UK; 2 Department of Pharmaceutical Sciences, University of Perugia, Via del Liceo 1, 06123 Perugia, Italy; 3 ICT Department, College of Science and Engineering, Hamad Bin Khalifa University, Doha, Qatar

**Keywords:** Drug-induced liver injury (DILI), Mechanistic models, Structural alerts, Protein target

## Abstract

**Background:**

Drug-induced liver injury (DILI) is a major safety concern characterized by a complex and diverse pathogenesis. In order to identify DILI early in drug development, a better understanding of the injury and models with better predictivity are urgently needed. One approach in this regard are in silico models which aim at predicting the risk of DILI based on the compound structure. However, these models do not yet show sufficient predictive performance or interpretability to be useful for decision making by themselves, the former partially stemming from the underlying problem of labeling the in vivo DILI risk of compounds in a meaningful way for generating machine learning models.

**Results:**

As part of the Critical Assessment of Massive Data Analysis (CAMDA) “CMap Drug Safety Challenge” 2019 (http://camda2019.bioinf.jku.at), chemical structure-based models were generated using the binarized DILIrank annotations. Support Vector Machine (SVM) and Random Forest (RF) classifiers showed comparable performance to previously published models with a mean balanced accuracy over models generated using 5-fold LOCO-CV inside a 10-fold training scheme of 0.759 ± 0.027 when predicting an external test set. In the models which used predicted protein targets as compound descriptors, we identified the most information-rich proteins which agreed with the mechanisms of action and toxicity of nonsteroidal anti-inflammatory drugs (NSAIDs), one of the most important drug classes causing DILI, stress response via TP53 and biotransformation. In addition, we identified multiple proteins involved in xenobiotic metabolism which could be novel DILI-related off-targets, such as CLK1 and DYRK2. Moreover, we derived potential structural alerts for DILI with high precision, including furan and hydrazine derivatives; however, all derived alerts were present in approved drugs and were over specific indicating the need to consider quantitative variables such as dose.

**Conclusion:**

Using chemical structure-based descriptors such as structural fingerprints and predicted protein targets, DILI prediction models were built with a predictive performance comparable to previous literature. In addition, we derived insights on proteins and pathways statistically (and potentially causally) linked to DILI from these models and inferred new structural alerts related to this adverse endpoint.

**Supplementary Information:**

The online version contains supplementary material available at 10.1186/s13062-020-00285-0.

## Background

Drug-induced liver injury (DILI) is a major safety concern and one of the leading causes of drug failure in clinical drug development and market withdrawal, which can be found across nearly all classes of medication [1]. DILI may occur either as hepatitis or cholestatic injury or a mixed form of both and can be further distinguished between intrinsic and idiosyncratic DILI [1]. If a drug is hepatotoxic in a dose-dependent manner both in preclinical models and humans (e.g. acetaminophen) it is considered to cause intrinsic DILI. Idiosyncratic DILI, on the other hand, is characterized by the lack of a clear dose-dependency and its rarity (usually less than 1 of 10,000 treated patients develops DILI symptoms). In contrast to intrinsic DILI, idiosyncratic DILI is the result of a patient’s rare combination of genetic and non-genetic risk factors, which is responsible for their susceptibility towards the drug [2]. Consequently, in most cases, idiosyncratic DILI cannot be detected in preclinical studies [3]. The idiosyncratic nature of DILI also impedes its prediction with quantitative structure-activity relationship (QSAR) models, as idiosyncrasy implies that the underlying cause lies beyond inherent compound properties. Due to the low incidence of DILI, revealing causal links between the use of a drug and an observed liver injury is a difficult task [4], which decreases the confidence in provided DILI labels and further complicates the building of QSAR models with high predictivity.

The limited capability of animal models to detect hepatotoxic compounds raises the need for alternative testing strategies including in vitro and in silico models, as well as a better understanding of the underlying biology. Major challenges associated with the prediction of DILI using in vitro approaches lie in identifying relevant assays [5] and extrapolating from assay concentrations to in vivo blood concentrations associated with a hepatotoxic risk [6]. Numerous in silico models have been generated based on molecular structure [7–13] and in vitro readouts, such as bioactivity [14], gene expression [15] in cell culture or combinations of readouts [16], which are able to predict DILI better than random, but with a performance not yet sufficient for decision making in practice.

In the case of computational predictions, DILI is often simplified to a classification problem, i.e. separating compounds with or without this annotation in a data set [7–9, 11, 14]. These labels, however, do not provide information on important factors such as dose-dependency or affected patient population, and consequently, the practical applicability of such models is limited. While more detailed information on quantitative compound toxicity is difficult to retrieve, the weight of evidence for DILI is often provided in the available datasets. Paying attention to the quality of the data used for model generation has previously been shown to be relevant; for example, Kotsampasakou et al. (2017) [9] demonstrated that better models can be derived with smaller, but higher quality datasets.

The present work is derived from participation in the Critical Assessment of Massive Data Analysis (CAMDA) “CMap Drug Safety Challenge” 2019 (http://camda2019.bioinf.jku.at) where the aim was to develop more predictive models for DILI from different descriptor spaces. In this study, we retrieved compound hepatotoxicity annotations from the DILIrank [17] and SIDER [18] databases which were used as labels to generate compound-based DILI classifiers. The annotations in DILIrank were assigned by considering DILI-related market withdrawals and warnings in drug labels in combination with assessing causal links between the use of the drug and the occurrence of DILI. The drug is annotated as “DILI positive” in two different severity classes (“vMost-DILI concern” and “vLess-DILI concern”) only if casual links to DILI could be confirmed. Drugs with existing concern but lack of causal proof were annotated as “Ambiguous DILI concern”, whereas drugs without concern were annotated as “vNo-DILI Concern”. The task set by the CAMDA challenge was to predict the labels of 55 drugs, which were previously annotated as “Ambiguous DILI concern” and recently re-classified by the FDA. To this end, multiple descriptors were derived from chemical structure which were used to build classification models for DILI: chemical fingerprints [19] describing the 2D compound structure, as well as Mordred molecular descriptors [20], and predicted protein targets inferred with PIDGIN [21–23]. The predictivity of the resulting models was evaluated using two different external test sets. Models were also built using the L1000 gene expression data provided by CAMDA, but these did not perform significantly better than random and were not analysed further (Additional file 1).

In addition to predictive performance, we also focused on two practically relevant aspects of DILI prediction, namely the ability of models to extrapolate in chemical space, as well as the interpretation of relevant molecular and biological factors underlying DILI since interpretable models are more trusted, for example by regulatory agencies [24]. To gain insights into biological processes, the protein targets with significantly higher binding probability in DILI compounds and the highest information for DILI classification were extracted from the protein target-based machine learning models. Based on these, we identified biological processes associated with DILI labels in the current dataset using genesets derived from MSigDB [25] to show that mechanistic understanding of the biology underlying DILI can be obtained from this chemical structure-based feature space.

From the purely chemical side, we derived interpretable structural alerts related to DILI with the Molecular Substructure miner algorithm (MoSS) implementation of graph-based Molecular Fragment miner algorithm (MoFa) [26] and the fragment-based SARpy package [27], which could guide lead optimization to reduce the risk of DILI, as is currently standard practice for other toxicities [28]. We then compared the quality of the derived structural alerts against the recent review of DILI related structural alerts by Liu et al. (2015) [29].

## Results

### Predictive modeling

We first compared the performance of Support Vector Machine (SVM) and Random Forest (RF) models trained using different input descriptors to predict DILI positive compounds. To this end, three datasets with differing levels of DILI label confidence and size were compared (summarized in Table 1): “DILIrank (−vLessConcern)”, comprising DILIrank compounds labelled as either vMostConcern or vNoConcern (high confidence), “DILIrank” which additionally contains compounds from the DILIrank vLessConcern class (low confidence), and “DILIrank (+SIDER)” which additionally includes inactives from the SIDER database (low confidence).

**Table 1:** Datasets used to generate predictive DILI models

Dataset Name	Data samples				Binary class	
	vLessConcern (*n* = 260)	vMostConcern (*n* = 174)	vNoConcern (*n* = 227)	SIDER (*n* = 262)	*n* (DILI)	*n* (NoDILI)
DILIrank (−vLessConcern)	–	DILI	NoDILI	–	174	227
DILIrank	DILI	DILI	NoDILI	–	434	227
DILIrank (+SIDER)	DILI	DILI	NoDILI	NoDILI	434	489

**Fig. 1:**
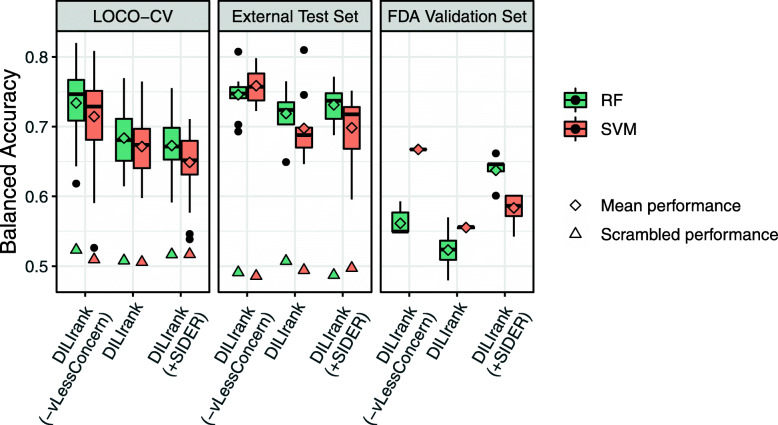
DILI label prediction performance (balanced accuracy) of RF and SVM models trained using ECFP4 descriptors. Models were trained using the datasets described in Table 1. Performance is stable between the 5-fold LOCO-CV and external test set, but a distinct drop in predictive accuracy is observed when predicting the FDA validation set. Hence, despite demonstrating a capability to generalize to new compounds (not seen during training) in the external test set, models lacked the capability to generalize to the new compounds in the FDA validation set

It can be seen in Fig. 1 that models trained using Extended Connectivity Fingerprints of diameter 4 (ECFP4) descriptors show similar predictive performance for both the Leave-One-Cluster-Out cross-validation (LOCO-CV) and the external test set across all datasets for both the RF and SVM algorithms. For example, RF models trained using the DILIrank (−vLessConcern) dataset had a mean balanced accuracy of 0.734 ± 0.044 during cross-validation and 0.746 ± 0.032 for the external test set (Table S1). Secondly, all models achieved higher prediction accuracy than y-scrambling models (Fig. 1), demonstrating they all had a predictive power exceeding that of pure chance [30]. Thirdly, for LOCO-CV and external test set a slightly better predictive performance was found using the highest confidence dataset in comparison to the lower confidence datasets, although it should be noted that these models are not directly comparable given the varying dataset sizes (Fig. 1 and Table 1). For example, for the SVM models the LOCO-CV mean balanced accuracy decreased from 0.714 ± 0.058 on the DILIrank (−vLessConcern) to 0.671 ± 0.043 (DILIrank) and 0.643 ± 0.045 (DILIrank (+SIDER)). Moreover, the mean external test set balanced accuracy decreased from 0.759 ± 0.027 (DILIrank (−vLessConcern)) to 0.697 ± 0.048 (DILIrank) and 0.709 ± 0.036 (DILIrank (+SIDER)). These three findings were also observed for models trained using Mordred molecular descriptors [20] and protein target descriptors [21, 22] (see Table S1, and Figs. S1 and S2).

A significant drop in performance is seen for the majority of models on the FDA validation set with a balanced accuracy of below 0.6 (Fig. 1), which indicated that the models were less capable of generalizing to the FDA validation set than to the external test set. These findings were also observed for models trained using Mordred molecular descriptors [20] and protein target descriptors [21, 22] indicating the limited generalization of models occurred irrespective of descriptor space. The best performing model across the most metrics was the SVM model trained using the DILIrank (−vLessConcern) dataset which utilized a linear kernel, a C parameter of 0.1, and a ‘one vs. rest’ decision function. This model achieved a mean balanced accuracy of 0.759 ± 0.03 and 0.655 ± 0.00 on the external test set and FDA validation set, respectively, thus demonstrating relatively high predictive power across the two independent test sets compared to all other models generated (Fig. 1 and Table S1).

**Fig. 2:**
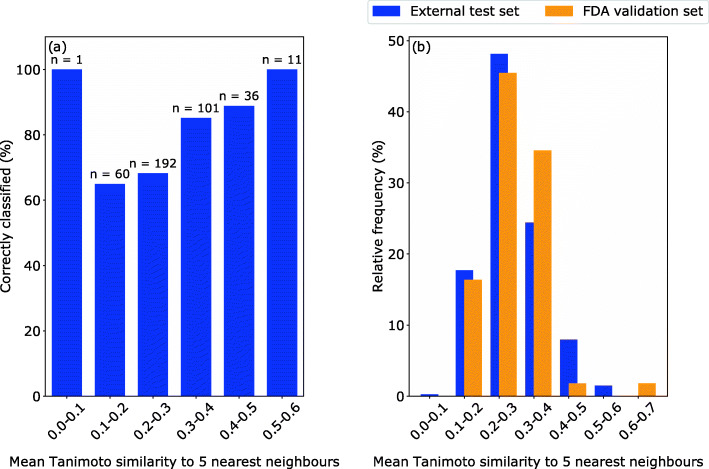
Analysis of the link between chemical similarity and classification performance. **a** Classification rate during LOO-CV vs. mean 5 NN Tanimoto compound similarity. A clear link between correct classification rate (%) and chemical similarity is observed in the DILIrank (−vLessConcern) dataset. The only exception from this was the first bin, which was defined by only a single compound (*n* = number of correctly classified compounds) and hence not a representative rate), and indicated that external test set compounds that are more structurally similar to the training set were predicted better. **b** Distribution of the mean 5 nearest neighbour inter-similarities between the DILIrank (−vLessConcern) training dataset and the corresponding test sets. It was found that the 55 FDA validation set compounds had comparable structural similarity to the training set (orange) as the compounds within the external test set (blue). Both histograms are left-closed

We next investigated the relationship between a compounds’ Tanimoto similarity to its 5 nearest neighbors in the training set and its classification performance for the external test set (Fig. 2a). This was achieved by generating a SVM model (with the same hyperparameters as the best model noted previously) where within each fold of a Leave-One-Out cross-validation (LOO-CV) scheme a compound’s predicted DILI label and Tanimoto similarity to the training set were retrieved (see Methods). Note that such an analysis for the FDA validation set was not possible as the DILIrank labels for this set of compounds were withheld. It was found that 65% of the compounds with a mean Tanimoto similarity to their 5 nearest neighbors in the training set between 0.0–0.2 were correctly classified (already comparable to the predictive performance on the FDA validation set for the same model - mean accuracy 0.673 ± 0.000), and this increased to 89% for compounds with 0.4–0.5 Tanimoto Similarity, and subsequently to 100% for compounds with a Tanimoto similarity greater than 0.5 (Fig. 2a). As similar inter-similarity distributions were found between the training dataset and both the external test and FDA validation sets (Fig. 2b), one could have naively anticipated a higher predictive performance (in line with the external test set) for the FDA validation compounds than seen in practice (Fig. 1).

### Biological interpretation of protein targets

**Fig. 3:**
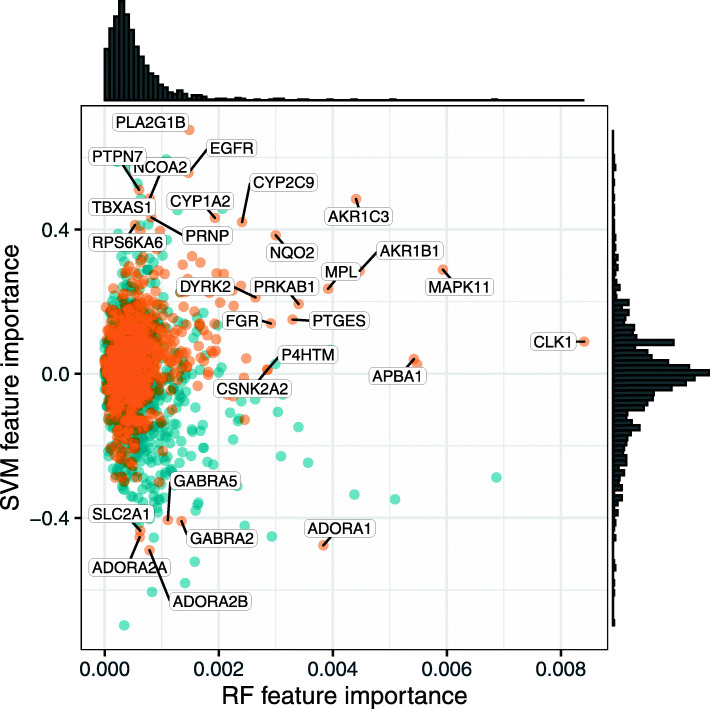
Distribution of protein feature importance in the best performing RF and SVM models. Proteins significantly enriched in the DILI class are labelled in orange, while all other proteins are colored in blue. Among the proteins with high feature importance are many whose involvement in DILI has been established before, such as AKR1B1, CYP1A2 and MAPK11, and this analysis might give further hints at novel proteins involved in DILI

We next compared the median feature importance of proteins in the RF and SVM model based on the DILIrank (−vLessConcern) dataset which showed the best classification performance with protein target descriptors across LOCO-CV, external test set and FDA validation set (Fig. S2). The Pearson correlation between the models' absolute respective feature importance is low (0.29) indicating that overall they identify different protein targets as being important for DILI classification. (Fig. 3)

Given that the focus is set on proteins with bioactivity related to DILI risk, we only further examined those that were significantly enriched in DILI-related compounds as determined by a Wilcoxon rank test. This included aldose reductase AKR1B1 which has been linked to APAP-induced oxidative stress and hepatotoxicity [31], the CYP enzymes CYP1A2 and CYP2C9 which are involved in xenobiotic metabolism in the liver [32], and the p38 kinase MAPK11 which is known to mediate stress-related signals in hepatotoxicity [33]. Moreover, aldo-keto reductase family 1 member C3 (AKR1C3) is essential for Phase II drug metabolism pathways and Transmembrane prolyl 4-hydroxylase (P4HTM) inactivation has reported to have a protective role against DILI [34–36].

However, novel proteins were also identified such as Dual specificity protein kinase (CLK1) and dual-specificity tyrosine-phosphorylation related kinase 2 (DYRK2). Interestingly, one of the identified novel proteins, namely Adenosine A1 Receptor (ADORA1), is a member of the same protein family as ADORA2A, which is known in liver damage [37, 38]. In fact, the adenosine receptors ADORA1 and ADORA2 share physiologic functions [39, 40] and ADORA1 has been found to contribute to renal dysfunction associated with acute liver injury in rats, supporting a plausible involvement of this target in DILI [41]. A full list of the proteins identified as containing the highest feature importance for classification of the current dataset with the RF and SVM methods and their known or potential links to hepatotoxicity are shown in Table S2.

**Fig. 4:**
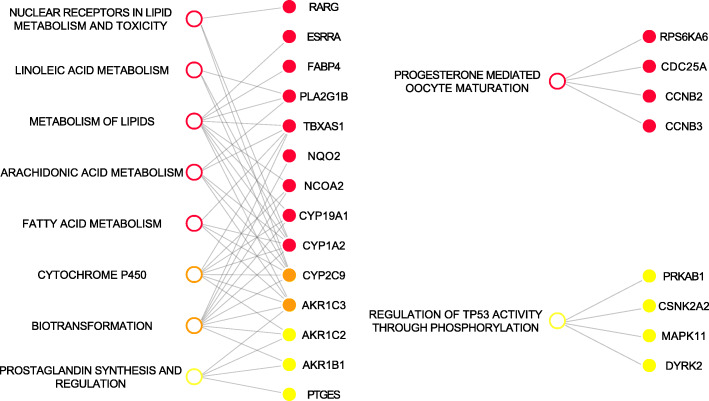
DILI-related processes inferred from predicted targets and pathway annotations. Processes were based on SVM (red) and RF (yellow) models or both (orange) on the DILI (−vLessConcern) dataset. All processes are linked to the corresponding over-represented proteins in grey (19 proteins with highest feature importance). Multiple highly similar genesets were combined to arachidonic acid metabolism, Cytochrome P450 and biotransformation with the individual genesets being mapped in Table S3. Biotransformation and Cytochrome P450 are identified by both methods, while additional pathways identified by SVM point to lipid metabolism and cell cycle, and TP53 regulation is identified by RF. Moreover, arachidonic acid metabolism (SVM) and prostaglandin synthesis (RF), two biologically closely related processes, are identified

In a next step, over-represented pathways were determined among the top protein targets, which were significantly enriched in the DILI positive compounds (false discovery rate (FDR) < 0.05) and showed high feature importance in either the RF or SVM models. While results across different feature importance thresholds are shown in Figs. S3 and S4, representative results of this analysis based on the 19 targets with the highest feature importance, respectively, are shown in Fig. 4. From both the RF and SVM models, biotransformation and Cytochrome P450 were identified as significantly overrepresented processes, each based on multiple genesets (see Table S3, Fig. S3 and Fig. S4), and the involvement of these two pathways in liver damage has been extensively characterized, especially for injuries related to drug metabolites [42–46]. Moreover, arachidonic acid metabolism and metabolism of lipids were retrieved by the SVM models, which play a well-established role in DILI, especially for injuries induced by acetaminophen [47, 48]. In contrast, RF identifies p53 signaling and prostaglandin synthesis as characteristic for DILI from the data (Fig. 4), which are key regulators of cellular stress response with a specific protective role against liver damages [49, 50]. Of note, prostaglandin synthesis and arachidonic metabolism are strictly related processes and have been identified by both RF and SVM at different feature importance thresholds (Fig. S3 and Fig. S4). In fact, prostaglandins are metabolites of arachidonic acid, whose production is controlled by cyclooxygenase (COX), which, in turn, is inhibited by NSAIDs, involved in DILI as stated above [51, 52]. Progesterone-mediated oocyte maturation is also over-represented in SVM and progesterone itself has a protective role against DILI [53]. More specifically, the proteins in this geneset point to cell cycle (M-phase inducer phosphatase 1 CDC25A, as well as the cyclins CCNB2 and CCNB3) and cell growth (RPS6KA6) with a reported role in DILI for Cyclin B2 CCNB2 [54]. Hence, both algorithms prioritize proteins known to be involved in key processes in DILI. The same analysis with the lower-performing models based on the DILIrank and DILIrank (+SIDER) datasets did not retrieve as many relevant proteins and pathways (results not shown).

### Structural alerts

**Fig. 5:**
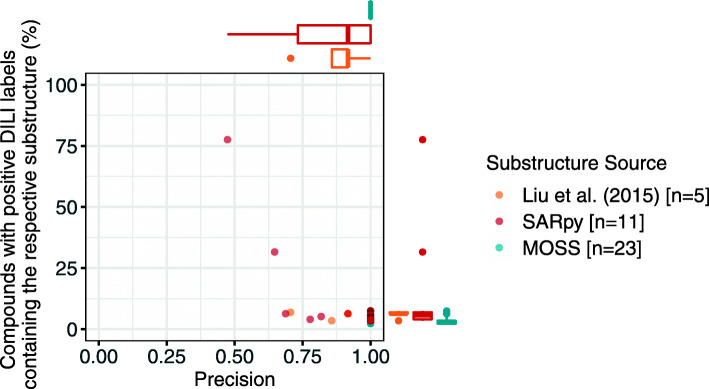
Precision and percentage coverage of significant DILI-related structural alerts (SAs) (*p*-value ≤0.05). The maximum coverage of SAs was 77.6% (benzene derivative generated by SARpy), however the average coverage was much lower - 6.68%. All MoSS-derived SAs had a precision of 1, the precision of SARpy SAs was lower on average but still relatively high - mean precision 0.85, and literature-derived alerts had a mean precision of 0.88

Two hundred thirty-three MoSS structural alerts (SAs) and 20 SARpy SAs were derived from the DILIrank (−vLessConcern) dataset, of which 23 and 11 were deemed significant (*p*-value ≤0.05), respectively. The number of derived SAs was sensitive to the parameters chosen and reflects the implementation of both algorithms (Methods). The quality of the inferred SAs, alongside 12 derived from the recent review literature of Liu et al. (2015) (5 of them were significant) [29], was assessed using multiple metrics (Table S4), with a particular focus on precision and coverage among compounds labelled as DILI positive (Fig. 5), with a summary of metrics per structural alert source in Table 2. Furthermore, an analysis of the occurrences of SAs in DrugBank [55] approved compounds was conducted.

**Table 2:** Metrics for DILI-related significant structural alerts (*p*-value ≤0.05)

Source	Mean precision	Mean coverage (%)	Min. compound presence in DrugBank	Max. compound presence in DrugBank
Liu	0.88 ± 0.11	6.09 ± 1.55	25	89
MoSS	1.0 ± 0.0	18.73 ± 10.73	3	41
SARpy	0.85 ± 0.18	14.11 ± 22.54	11	1400

Mean precision and coverage (%) of significant structural alerts from MoSS, SARpy, and Liu et al. (2015) indicated that highly precise SAs alerts were generated from all three sources, but that these observed varying degrees of coverage. The minimum and maximum presence of SAs for each source in DrugBank approved compounds demonstrated that all SAs were found in at least some approved compounds, but that the absolute frequency varies significantly between SAs

Overall, for both data- and literature-derived SA a common trade-off between precision and coverage was observed i.e. if a substructure had a high precision it rarely had a high coverage (Fig. 5). For example, the SAs benzene derivative (SARpy) and aniline derivatives (SARpy) had relatively low precision (0.47 and 0.65) relative to the mean precision of 0.85 ± 0.18 for SARpy SAs, but conversely had relatively high coverage (77.59 and 31.61%) relative to the mean coverage of 14.11 ± 22.54% for SARpy SAs. Table 2 affords a comparison between the precision and coverage of SA from each of the three sources analyzed in this study. In particular, it was noted that the SAs extracted by Liu et al. [29] had lower coverage than those generated by MoSS and SARpy, but that SAs from all sources had high precision on average. Furthermore, it was seen that SAs from all methods were found in at least some approved compounds from the DrugBank database.

A maximum precision of 1 occurred for 29 out of 39 of the significant SA, of which hydrazine derivative was seen for all three sources and had the highest coverage in DILI positive compounds (7.47%). Thus, despite the fact, for example, that SARpy could generate SAs with a precision of 1, the analyzed SAs were overly specific and only occurred in a very small proportion of DILI positive compounds.

## Discussion

### Predictive modeling

Overall, the most predictive model generated in this study was an SVM model (linear kernel, C = 0.1, ‘one vs. rest’ decision function) trained using the DILIrank (−vLessConcern) dataset, and ECFP4 chemical structure descriptors. In contrast to non-linear models such as those generated using RF, the linear kernel utilized by this SVM model ensured a lack of interaction between input variables which may have facilitated the model’s improved generalization properties. This model ranked third and first for cross-validation (0.714 ± 0.058) and external test set (0.759 ± 0.03), respectively, by balanced accuracy, across a compendium of studies that sought to develop classification models for DILI, despite the significantly smaller dataset used for model training in this study (Table 3). It should be noted that datasets, features, and cross-validation schemes used across these studies vary and therefore performance metrics can hardly be compared directly. Among others, a LOCO-CV scheme was implemented in the present study to avoid predicting compounds with high Tanimoto similarity to the training data, which is more conservative than the random splits used by Kotsampasakou et al. [9] (Table 3) and translated to a more rigorous evaluation of internal model performance.

**Table 3:** Comparison of performance indicators to several DILI classification models reported in the literature

Model algorithm	Number of Compounds	CV scheme	CV Balanced accuracy	CV Sensitivity	External test set Balanced accuracy	External test set Sensitivity	References
RF	996 (541+/455-)	10-fold, random splits	0.645	0.680	0.588	0.536	Kotsampasakou et al. (2017) [9]
SVM	1317 (571+/407-)	5-fold, splitting scheme unknown	0.767	0.948	0.597	0.848	Zhang et al. (2016) [10]
Ensemble of RF and SVM models (5 total)	1241 (683+/558-)	5-fold, splitting scheme unknown	0.701	0.799	0.719	0.909	Ai et al. (2018) [7]
Ensemble of eight different algorithms derived models (8 total)	1254 (636+/618-)	10-fold, splitting scheme unknown	0.783	0.818	0.716	0.773	He et al. (2019) [8]
SVM	401 (174+/227-)	5-fold, Tanimoto similarity based GroupKFold	0.714 ± 0.06	0.697 ± 0.08	0.759 ± 0.03	0.724 ± 0.08	Present study

Literature model performance derived from He et al. (2019) [8]. External test values quoted for the model developed in the present study are for the external test set. Despite being trained on the fewest compounds (401) and using a conservative LOCO-CV cross-validation scheme, the SVM model developed in the present study demonstrated robust predictivity between cross-validation and external test set

Two key trends relating to training data quality and model bias were identified. Firstly, a large proportion of models, irrespective of descriptor type, showed consistent performance between cross-validation and the external test set, but then observed a steep drop in performance on the FDA validation set (Figs. 1, S1, and S2) despite showing similar chemical similarity distribution to the training set as seen for the external test set (Fig. 2b). One explanation for this is that both test sets populate different regions of chemical space with the model showing better performance in one area, or that while populating similar regions of chemical space, these are not clearly attributed to one of the DILI classes and intrinsically difficult to predict. An alternative explanation for the lack of generalization could also be attributed to the fact that compounds in the training data and external test set were labeled with higher confidence and hence the model might be able to distinguish between DILI positive and DILI negative compounds well. In contrast, the compounds of the FDA validation set, originally being labeled as ambiguous due to lack of clear evidence, might be inherently more difficult to predict. Deriving accurate DILI labels for compounds is a complex process given the uncertainty of causality assessment and the difficulty in trying to incorporate administration factors such as dose and patient populations. Moreover, phenomena such as idiosyncratic DILI which cannot usually be detected even in preclinical studies and occur only in subpopulations make the task of accurate DILI labeling even harder [3].

Secondly, across all descriptor types (ECFP4, Mordred molecular descriptors, and predicted protein targets) balanced accuracy in the cross-validation and on the external test set decreased as the training dataset was expanded from the high confidence dataset (DILIrank (−vLessConcern)) to either of the lower confidence datasets - DILIrank or DILIrank (+SIDER) (Figs. 1, S1, and S2). This indicated that the inclusion of compounds from the vLessConcern class from DILIrank i.e. those with lower annotated evidence for DILI risk, as well as inactives derived by text-mining of package label inserts of marketed drugs (SIDER), harmed predictive performance despite increasing the number of training samples. While this is consistent with previous studies [9] which demonstrated that careful data curation can lead to improved performance, it should be noted that the sample size of the external test set and in particular the FDA validation set (49 compounds) were small. This makes it difficult to accurately evaluate model performance and accordingly also to confidently compare models (Fig. 1).

Larger datasets would be required to allow for enhanced fine-grained sampling of chemical space and the establishment of a model applicability domain. In the present study, the poor generalization to the FDA validation set demonstrated that the relationship between chemical structure and the propensity to cause DILI is too complex for the model to learn from the small training dataset used (401 compounds). However, it must be noted that even if larger and higher quality datasets were acquired, model predictivity would still be limited as relevant information that may relate to the manifestation of DILI such as dose or the influence of metabolism in the formation of hepatotoxic prodrugs were not considered in the descriptors used in the present study.

### Protein targets

From the models which used predicted protein targets as features, we extracted biological processes by incorporating prior knowledge on bioactivity using PIDGIN and the functional contexts of proteins based on pathway maps from multiple databases derived from MSigDB [25]. SVM and RF both identified biotransformation and Cytochrome P450, two important pathways involved in drug metabolism and elimination and strictly related to DILI [42–46]. Moreover, arachidonic acid metabolism and prostaglandin synthesis are identified, which are physiologically involved in the inflammation process [49, 50] and the mechanism of action and toxicity of NSAIDs, one of the most common causes for DILI [51, 52]. While the inferred biological processes have been known to be associated with DILI, this is not true for many of the proteins identified by feature importance themselves (Table S2), such as CLK1 and DYRK2. Given that the analysis was based on target binding probabilities, it can be hypothesized that these proteins might be off-targets directly (or indirectly) involved in the pathogenesis of DILI. The described workflow hence was able to derive functional hypotheses on biological processes from compound DILI annotations, which can subsequently be investigated experimentally.

### Structural alerts

In this study, structural alerts (SAs) related to DILI were derived using the SARpy [27] and MoSS [26] algorithms using the DILIrank (−vLessConcern) dataset. Both MoSS and SARpy derived SA were found to be comparable to those reviewed by Liu et al. (2015) [29] in terms of precision and coverage. It should be noted that in contrast to the SA of SARpy and MoSS which were explicitly derived and subsequently tested on the dataset used in this study, the SA of Liu et al. (2015) were derived using data from different sources, mainly LiverTox [56].

Of the significant SA obtained by SARpy, MoSS, and Liu et al. (2015) only hydrazine derivative (NN) was found to overlap between all of them (Table 4) and this obtained a precision of 1. However, a DrugBank [55] database search of the significant SA showed that all of the significant SA derived using MoSS occurred in at least 3 approved drugs, and those from SARpy and by Liu et al. (2015) occurred in at least 10 approved drugs (Table S4). For example, aniline derivative (SARpy) and carbamide derivative (SARpy), were present in 422 and 80 marketed drugs, respectively (Table S4). From the methodological angle it illustrated that whilst SA can be informative about an increased probability of a compound being toxic, the presence of all structural alerts analyzed in this study in DrugBank approved compounds demonstrated they are not diagnostic of DILI in isolation. Administration dose is a key consideration to make when developing therapeutics and is not taken into account when simply screening for the presence of a structural alert. For example, hydrazine derivatives (shared between SARpy, MoSS, and Liu et al. (2015)) can increase muscle, neural, kidney, liver, blood and spleen toxicity [57], however, it is present in e.g. procarbazin, which is a registered antineoplastic agent used in Hodgkin’s disease treatment and is an orphan drug for glioma [58]. This example demonstrates that it can be beneficial to accept an increased toxicity risk in favor of prolonging the patient’s life.

**Table 4:** DILI-related significant structural alerts (*p*-value ≤0.05) with the highest precision and coverage (%)

Source	Substructure	Name	Precision	Coverage (%)	*p*-value	Presence in DrugBank	DrugBank compound with SA
Liu, SARpy, MoSS		Hydrazine derivative (NN)	1	7.47	0.00001	37	testosterone enantate benzilic acid hydrazone
MoSS		Hydrazine derivative (N(−N)-C)	1	6.32	0.00009	33	testosterone enantate benzilic acid hydrazone
SARpy		Furan derivative (c1ccco1)	1	5.75	0,0002	19	diloxanide furoate

The following quality metrics are shown for the best substructures from MoSS, SARpy, and Liu et al. (2015): Precision, coverage in DILI positive compounds (%), *p*-value, as well as the number of approved compounds in Drugbank [44] which contain the substructure, alongside an example of such a structure

SAs can play a supportive role in initial screening and exploratory analysis by flagging potentially toxic compounds early [59, 60] and guiding lead optimization by medicinal chemists [61]. Their main advantage is that they are easy to understand and implement [62]. However, one should be cautious when interpreting frequency analysis results in the case of complex endpoints as SAs might not capture sufficiently the underlying biological mechanisms resulting in high false positive and false negative rates [63].

## Conclusions

In this study, DILI classifiers were trained using data from the DILIrank and SIDER databases, by employing the SVM and RF algorithms with either ECFP4 fingerprints, Mordred molecular descriptors, or predicted protein targets as chemical structure-derived descriptors. The best predictive performance was seen when using more reliable data (excluding the DILIrank vLessConcern class and SIDER text-mined inactives). This underlines the importance of data quality for such approaches, although it should be noted that a true comparison is difficult given the difference in size between datasets, and the generally small number of samples. The best model achieved comparable performance in cross-validation and on the external test set to models reported in the literature (Table 3). On the other hand, performance on an additional test set provided by CAMDA (http://papers.camda.info/) was much lower, underlining the difficulty of accurately validating DILI models given the low number of labeled compounds. In the present study, the datasets used to evaluate the DILI model were both small, consisting of 80 (for the high confidence dataset) and 49 compounds respectively, with the latter proving much more difficult to predict despite the comparable structural similarities of the two datasets to the model training dataset.

Protein target descriptors achieved inferior predictive performance, but their advantage, compared to Mordred molecular descriptors and ECFP4 fingerprints, is that each individual feature corresponds to a protein and is hence interpretable from a biological perspective. Based on the feature importances in predictive models, it was hence possible to identify known and potentially novel key proteins involved in DILI, as well as important biological processes in drug-induced liver apoptosis, such as biotransformation and the mechanism of action and toxicity of NSAIDs, which are known to be a common cause for DILI.

Moreover, we inferred structural alerts with comparable precision and coverage to previously derived ones. However, due to the high structural diversity of DILI annotated compounds, the derived alerts were found to have rather low compound coverage by themselves. Moreover, all alerts were found to be present in approved drugs further highlighting the challenge in deriving practically useful structural alerts for DILI, and underlining the importance of quantitative factors such as dose when screening compounds for DILI. Hence, overall, this work achieved similar results as seen in previous studies with respect to performance of predicting DILI; on the other hand it introduced the utilization of biologically interpretable predicted protein targets to the field and underlined the importance of large and reliable dataset annotations when developing predictive models for DILI.

## Methods

### Data preparation

Compounds’ SMILES strings were retrieved from the DILIrank database (1036 compounds) [17], and the SIDER 4.1 database (1430 compounds) [18]. Side effects are recorded in SIDER using the preferred terms of the MedDRA (Medical Dictionary for Regulatory Activities), which provides a hierarchical organization of adverse events. Starting with the entire SIDER dataset (SIDER 4.1), all compounds with at least one reported side effect contained in the MedDRA’s System Organ Class hepatobiliary disorders were discarded to keep only drugs for which no liver-related side effects have been reported. The SMILES retrieved from DILIrank and SIDER were standardized using the Python package Standardiser of Atkinson et al. (2016) [64]. This involved the removal of counterions and solvents and the neutralization of the remaining fragments if necessary. Moreover, tautomers were standardized according to the rules implemented in the standardizer. Subsequently, compounds that fell into at least one of the following categories were discarded: mixtures of more than one active ingredient, inorganic molecules, metal-organic compounds, and compounds with a molecular weight above 1 kDa. If compounds were present in both the DILIrank and the SIDER dataset, the compound from the SIDER inactive dataset was removed to avoid duplicate entries. The final set contained 923 compounds composed as follows: DILIrank: 174 vMost-DILI-Concern, 260 vLess-DILI-Concern, 227 vNo-DILI-Concern, SIDER: 262 compounds without reported liver-related side effects.

ECFP4 [17] hashed to 2048 bits were generated using the Python library RDKit (version 2019.03.1.0) [65]. One thousand one hundred eighty-nine 1D and 2D molecular descriptors were generated using the Python package Mordred [20]. For the generation of models, the values of the molecular descriptors were scaled to a Gaussian distribution with zero mean and unit variance using the *StandardScaler* function in the scikit-learn Python library (version 0.21.2) [66]. Bioactivity for 1673 human protein targets was predicted using the PIDGINv3 software [21–23]. 10 μM was chosen as the bioactivity cut-off to consider highly and marginally active compounds. To get a prediction for every compound-target pair, no threshold for the applicability domain was applied. For 6 out of the 923 drugs (4 of them from SIDER) no protein target prediction was made, since their structures could not be standardized internally in the PIDGINv3 software.

In order to implement a LOCO-CV scheme (to ensure similar compounds were not in different folds), we performed hierarchical clustering of compounds. Based on pairwise Tanimoto similarities calculated using ECFP4, a tree was generated using hierarchical clustering with the Nearest Point Algorithm implemented in SciPy (version 1.2.1) [67]. Clusters were generated by cutting the hierarchical tree at a distance of 0.5, which resulted in compounds with a Tanimoto similarity of at least 0.5 being in the same cluster.

### Model generation

#### Overview

We chose SVM and RF as methods as they have demonstrated good and robust performance, and are less prone to overfitting in comparison to more sophisticated methods. For both methods, we used the scikit-learn Python library (version 0.21.2) implementations to train binary classification models for DILI. Models were developed for all three input feature spaces (ECFP4 fingerprints, protein targets, and Mordred molecular descriptors). In addition, we generated models using different subsets of data considering different DILIrank classes as well as additional inactives from the SIDER database (Table 1).

#### Model Hyperparameter grid search

Firstly, for SVMs [68] we used a classifier as implemented in the sklearn Python library and performed a hyperparameter grid search over the following parameters: Kernel: [‘linear’], Class weight: [‘balanced’], Decision function: [‘one vs. rest’], Shrinking: [‘True’], C: [0.05, 0.1, 0.2, 0.3, 0.4, 0.5, 1]. Of the possible SVM kernels implemented sklearn, we only evaluated ‘linear’ as it alone allows for easy interpretation of model feature importances. Secondly, for RFs [69] we used a classifier as implemented in the sklearn Python library and performed a hyperparameter grid search over the following parameters: Bootstrap: [‘True’], Class weight: [‘balanced subsample’], Max. Tree depth: [10, 15, None], Min. samples per leaf: [1–3], Number estimators: [100, 200, 300, 400, 500, 750, 1000].

#### Training procedure

The predictive performance of models was evaluated using a 5-fold cross-validation inside a 10-fold training scheme. The original dataset was split into 10 stratified folds based on DILI class label using the StratifiedKFold function in scikit-learn with parameters: n_splits = 10, and shuffle = True to assess the impact of different training data on model predictive performance. Within each training fold, an internal grid search over model hyperparameters was conducted using an internal 5-fold LOCO-CV to select the best model per training fold. The best model in each fold, assessed by balanced accuracy, was then used to predict for the holdout external test set. The LOCO-CV scheme was implemented with the GroupKFold function in scikit-learn with clusters only containing compounds with a ECFP4 Tanimoto similarity [70] greater than or equal to 0.5. This cross-validation scheme was utilized irrespective of the descriptor type. In addition, baseline models were trained and evaluated using the same procedure as mentioned, but with prior y-scrambling of the output labels (3 runs of different random scramblings). To further evaluate model predictive performance, an FDA validation set composed of 49 compounds (a subset of 55 - the CAMDA organizers removed 6, of which were unknown to the authors) previously labeled as vAmbiguous-DILI-Concern, but later relabeled as DILI positive or DILI negative by the FDA was used as an additional test set.

To evaluate the relationship between the mean chemical similarity of a compound to its 5 nearest neighbors in the training set and model correct classification rate a LOO-CV was conducted for the best performing model (SVM, DILIrank (−vLessConcern, (Fig. 2a). This required the calculation of the Tanimoto similarities between the training dataset and the left-out compound using ECFP4 fingerprints and predicting its DILI label within each LOO-CV fold. Furthermore, the Tanimoto 5 nearest neighbor inter-similarity of the FDA validation set to the training set was compared to the corresponding similarities of the external test set (Fig. 2b). As we previously evaluated the performance of the model (SVM, DILIrank (−vLessConcern)) with 10 distinct external test sets (in a 10-fold cross-validation scheme; see above), the similarities are averaged across all 10 pairs of training and test set.

Balanced accuracy (eq. 1) was primarily used to assess the predictive performance of the models. We also utilized specificity to compare models to those previously published in the literature (Table 3). Those metrics were calculated from a confusion matrix consisting of true positives (TP), true negatives (TN), false positives (FP) and false negatives (FN).

1 $$ \mathrm{Balanced}\ \mathrm{Accuracy}=\frac{\frac{TP}{\left( TP+ FN\right)}+\frac{TN}{\left( TN+ FP\right)}}{2} $$

### Interpretation of protein targets

Protein targets with higher binding probability in DILI positive compounds, called DILI-enriched targets, were determined using a one-sided Wilcoxon rank-sum test with a FDR of < 0.05. Among those, proteins features with high median importance across the 10 train-test splits were identified for RF and SVM models, respectively. The feature importance for RF models implemented in scikit-learn [66] describes the decrease of node impurity achieved by a feature, averaged over all trees in the forest, as a fraction, so that the importance of all features included in the model sum up to 1 [69]. In SVM models using linear kernels the importance of features is reflected by the magnitude of their coefficients describing the hyperplane [68]. The sign indicates which class is favored by the presence of a given feature. The values used for further analysis were the median importance of a feature across the 10 train-test splits.

Over-enrichment analysis was performed using the clusterprofiler R package (version 3.17.5) [71]. For this, pathway maps were derived from MSigDB [25] via the msigdbr R package from Reactome [72], KEGG [73] and Wikipathways [74]. To this end, the protein targets with highest feature importance were mapped to Entrez gene IDs with the biomaRt package [74] and the list of PIDGIN target proteins was used as background. Only gene sets containing 10 or more genes were considered and *p*-values were adjusted using the Benjamini-Hochberg procedure. The analysis was performed using various feature importance thresholds scanning across the top quantile of absolute feature importance values.

### Structural alerts

#### Derivation of structural alerts

Two algorithms for SA derivation were used, with the DILIrank (−vLessConcern) dataset as input - MoSS and SARpy. MoSS is a graph-based depth-first search method used for chemical substructure mining [26] and we used the KNIME (v3.7.2.) [61] implementation of MoSS in the current study. It derives potential SAs as “subgraphs” with only heavy atoms, which are neither SMILES nor SMARTS. Users might decide to approximate the subgraphs with SMARTS in order to match the substructure to molecules (denoted by SMILES). This program searches for frequent molecular substructures and discriminative fragments in a set of molecule graphs. In a graph, a vertex is a representation of an atom and an edge is a representation of a bond. Each vertex has attributes related to atom type, charge, and whether it is a part of an aromatic ring. Edges indicate the bond type. The search starts from the root of the graph tree being a single atom and follows recursively through atoms linked to leaf atoms with subsequent bonds. Substructures are then created based on each state of the graph tree and are pruned if the substructure occurrence in the active class is lower than the defined minimum focus support (MFS).

In order to find a discriminative fragment, two thresholds should be defined by the user. The first one is the aforementioned MFS used for pruning and the second one is minimum complement support (MCS) i.e. the substructure occurrence in the inactive class. The following KNIME MoSS settings were chosen: 1% MFS (the minimum fraction of the fragment-contained chemicals in the DILI positive class - the true positive rate), 0.01% MCS (the maximum fraction of the fragment-contained drugs in the DILI negative class - the false positive rate). In addition, only substructures in which the number of bonds ranged from 2 to 15 were kept. Pure carbon fragments were ignored and ring mining was applied.

SARpy is a string-based search method used for chemical substructure mining [27]. Briefly, SAs in the form of SMARTS strings are generated by recursively breaking every combination of bonds working directly on the SMILES strings of the input dataset. Fragments are then internally validated against all compounds in the dataset, and then a reduced set of substructure “rules” is extracted. In this work’s implementation of SARpy (v.1.0) the *fragmentize function* parameters minAtoms and maxAtoms were set to 2 and 15 respectively, and the ‘target’ (i.e. DILI positive or DILI negative) was set to None. Structural alerts for DILI were extracted using the *extract function* with the parameters: 5 minHits, 1 minLR, and 0 minPrecision. These settings are identical to those used by Yang et al. (2017) [75], except that a precision threshold was not applied in order to generate a larger compendium of SAs to analyze.

### Evaluation of structural alerts

Structural alerts’ SMARTS were matched to compounds’ SMILES using the RDKit *HasSubstructMatch function* in Python (RDKit 2018.09.3.0). Precision (eq. 2) and coverage in DILI positive compounds were both used to assess the predictive performance of the SAs. In addition, significance measured by *p*-value was also calculated for each SAs using the SciPy (version 1.3.0) stats module *fisher_exact function* with alternative parameter set to ‘greater’.

2 $$ \mathrm{Precision}\ \left(\mathrm{P}\right)=\frac{TP}{\left( TP+ FP\right)} $$

As previously mentioned, because MoSS utilizes a graph-based search approach it may not consider the slight difference between aromatic and aliphatic atoms, leading to mismatches when matching its substructures to SMILES. For example, in MoSS, “N-C” can match both aminofuran (NC1 = CC=CO1) and aminotetrahydrofuran (NC1CCCO1). However, in SMARTS, “C” is different from “c”, so RDKit will not match “N-C” with aminofuran, because the carbons are aromatic. Despite this, the significance of these SAs is based on the presence calculated using RDKit.

To investigate a SA’s presence in already approved and marketed drugs, SAs were matched to compounds in the DrugBank database [55] (v.5.1.4) using the RDKit (version 2018.09.1) [65] *HasSubstructMatch* function. This involved firstly standardizing compounds’ SMILES using the Python package Standardiser of Atkinson et al. (2016) [64]. As some SMILES could not be standardized, this step reduced the total number of DrugBank compounds in the analysis from 2411 to 2136.

## Supplementary Information

**Additional file 1: SI.** Overview of gene expression data preparation, DILI model generation, and DILI label prediction performance for models derived using L1000 gene expression data. Gene expression data for 14 distinct cell line-time-dose combinations was extracted for all compounds with DILI labels. Replicate measurements for the same compound were not aggregated resulting in differing numbers of positive and negative data points for each dataset. Separate RF and SVM classification models were trained using either the DILIrank or the DILIrank (−vLessConcern) datasets for each of the 14 distinct cell line-time-dose combinations. Unlike models generated using descriptors derived from chemical structure, the RF and SVM models developed did not achieve meaningfully higher prediction accuracies than y-scrambling models.

**Additional file 2: Figure S1.** DILI label prediction performance (balanced accuracy) of RF and SVM models trained using the DILIrank (−vLessConcern) dataset and Mordred molecular descriptors for 5-fold LOCO-CV, external test set, and FDA validation set (Methods). The balanced accuracy for 5-fold internal cross-validation, external test set, and FDA validation set for 10 models trained using different training data sets (DILIrank (−vLessConcern), DILIrank, DILIrank (+SIDER)) and training dataset splits is shown via whisker plots. The median model performance of 3 y-scrambled models is shown as triangles for cross-validation and external test set. Predictive accuracy is stable between cross-validation and external test set, but a distinct drop in predictive accuracy is observed when predicting the FDA validation set.

**Additional file 3: Figure S2.** DILI label prediction performance (balanced accuracy) of RF and SVM models trained using the DILIrank (−vLessConcern) dataset and protein target descriptors for 5-fold LOCO-CV, external test set, and FDA validation set (Methods). The balanced accuracy for 5-fold internal cross-validation, external test set, and FDA validation set for 10 models trained using different training data sets (DILIrank (−vLessConcern), DILIrank, DILIrank (+SIDER)) and training dataset splits is shown via whisker plots. The median model performance of 3 y-scrambled models is shown as triangles for cross-validation and external test set. Predictive accuracy is stable between cross-validation and external test set, but a distinct drop in predictive accuracy is observed when predicting the FDA validation set.

**Additional file 4: Figure S3.** Enriched pathways across different feature importance cutoffs for RF using the DILIrank (−vLessConcern) dataset. Enriched pathways are shown across different feature importance cutoffs which are identified by the percentile of DILI-enriched protein targets covered. Significant pathways (FDR < 0.05) are colored by -log (FDR), pathways without any gene present are shown in white and insignificant ones in grey. Regulation of TP53 through phosphorylation is the pathway conserved at the highest threshold identifying significant pathways. Other identified pathways include arachidonic acid metabolism and prostaglandin synthesis.

**Additional file 5: Figure S4.** Enriched pathways across different feature importance cutoffs for SVM using the DILIrank (−vLessConcern) dataset. Enriched pathways are shown across different feature importance cutoffs which are identified by the percentile of DILI-enriched protein targets covered. Significant pathways (FDR < 0.05) are colored by -log (FDR), pathways without any gene present are shown in white and insignificant ones in grey. While some pathways are only significant at high thresholds, such as steroid hormone biosynthesis, others are only found at lower thresholds, e.g. TLR signaling. Additionally, a set of pathways including biotransformation, cytochrome 450 and arachidonic acid metabolism are observed across the majority of thresholds.

**Additional file 6: Table S1.** Performance of models trained using the DILIrank (−vLessConcern) dataset. Shown is mean ± standard deviation for 7 metrics (MCC - Matthew’s Correlation Coefficient, PRAUC - Precision-Recall Area Under Curve, ROCAUC - Receiver-Operator-Characteristic Area Under Curve) for models trained using ECFP4, Mordred molecular descriptors (MD), and protein target descriptors (PT). Row names correspond to the descriptor type, algorithm, and the test set - external test set (ET), FDA validation set (FDA). The best external test set and FDA validation set performance per metric are shown in **bold**. For the FDA validation set, PRAUC and ROCAUC were not available as only the confusion matrices of the predictions were provided by CAMDA. The model trained using SVM and ECFP4 descriptors achieved the best performance over the FDA validation set.

**Additional file 7: TableS2.** Proteins with high feature importance in RF and SVM, and links to DILI. The 19 proteins with the highest feature importance in RF or SVM models are shown. The feature importance is shown in bold if the protein ranked among the top 19 in the respective model. Many proteins identified possess known functions in liver drug metabolism and cell stress. Those proteins with plausible involvement in DILI are indicated in italics.

**Additional file 8: TableS3.** Pathways with high feature importance in RF and SVM, and links to DILI. The overrepresented gene sets for 19 proteins with the highest feature importance in RF or SVM models are shown. Many pathways identified possess known functions in liver drug metabolism and cell stress.

**Additional file 9: TableS4.** Top significant structural alerts (*p*-value ≤0.05). The following quality metrics are shown: precision, coverage in DILI positive compounds (%), and number of Drugbank [44] approved compounds with the substructure are shown. *MoSS substructure notation is in the form of subgraphs with only heavy atoms, which are neither SMILES nor SMARTS.

## Data Availability

The datasets generated and analyzed during the current study are available in the GitHub repository https://github.com/anikaliu/CAMDA-DILI.

## Abbreviations

ADORA1: Adenosine A1 receptor
AKR1C3: Aldo-keto reductase family 1 member C3
CAMDA: Critical assessment of massive data analysis
CLK1: Dual specificity protein kinase CLK1
DYRK2: Dual-specificity tyrosine-phosphorylation related kinase 2
DILI: Drug-induced liver injury
ECFP4: Extended connectivity fingerprints of diameter 4
FDR: False discovery rate
FN: False negative
FP: False positive
TN: True negative
TP: True positive
LOCO-CV: Leave-one-cluster-out cross-validation
LOO-CV: Leave-one-out cross-validation
MCS: Minimum complement support
MedDRA: Medical dictionary for regulatory activities
MFS: Minimum focus search
MoFa: Molecular fragment miner algorithm
MoSS: Molecular substructure miner algorithm
NASH: Non-alcoholic steatohepatitis
NSAIDs: Nonsteroidal anti-inflammatory drugs
P4HTM: Transmembrane prolyl 4-hydroxylase
QSAR: Quantitative structure-activity relationship
RF: Random forest
SA: Structural alert
SVM: Support vector machine
