# Supplementary material for: Prediction and mechanistic analysis of drug-induced liver injury (DILI) based on chemical structure
Source: Biol Direct. 2021 Jan 18;16:6. doi: 10.1186/s13062-020-00285-0 (PMC7814730; doi:10.1186/s13062-020-00285-0)
Supplement: Supplementary file 1 — Additional file 1: SI. Overview of gene expression data preparation, DILI model generation, and DILI label prediction performance for models derived using L1000 gene expression data. Gene expression data for 14 distinct cell line-time-dose combinations was extracted for all compounds with DILI labels. Replicate measurements for the same compound were not aggregated resulting in differing numbers of positive and negative data points for each dataset. Separate RF and SVM classification models were trained using either the DILIrank or the DILIrank (−vLessConcern) datasets for each of the 14 distinct cell line-time-dose combinations. Unlike models generated using descriptors derived from chemical structure, the RF and SVM models developed did not achieve meaningfully higher prediction accuracies than y-scrambling models. [file 13062_2020_285_MOESM1_ESM.pdf]

Predicting DILI from gene expression profiles

LINCS gene expression signatures provided by CAMDA (http://papers.camda.info/) ('CAMDA_l1000_1314compounds-GSE92742_Level5_gct.rda') were processed in RStudio (version 1.1.463) running R (version 3.5.2). 14 cell line-time-dose combinations with gene expression signatures for all compounds with DILI labels were identified and expression of the 978 directly measured landmark genes extracted. Moreover, expression profiles on compounds with the DILI class vAmbiguous-DILI-Concern were separated out. Replicate signatures i.e. where the same compound was tested multiple times in the same cell line-time-dose condition were not aggregated; this resulted in differing numbers of data points per experimental condition as seen below in Table SI_GEX 1. We also generated a combination dataset consisting of data from all experimental conditions which is denoted as 'All cell lines'.

Table SI_GEX 1. Datasets used to generate predictive DILI models using LINCS gene expression data (landmark genes only). Although all cell lines contain expression profiles on all training compounds, the number of data points varied between datasets as each contained different numbers of replicates. The number of overall data points is shown for each dataset and the number of compounds indicated per DILI class.

Dataset Name	Data points		
	vLessConcern (n=90)	vMostConcern (n=37)	vNoConcern (n=51)
A375, 6 h, 10 uM	240	94	144
A549, 24 h, 10 uM	172	63	129
ASC, 24 h, 10 uM	109	47	70
HA1E, 6 h, 10 uM	193	77	121
HCC515, 6 h, 10 uM	150	67	107
HEPG2, 6 h, 10 uM	172	64	104
HT29, 6 h, 10 uM	238	94	142
MCF7, 6 h, 10 uM	335	124	201
MCF7, 24 h, 10 uM	290	111	202
PC3, 6 h, 10 uM	236	89	149
PC3, 24 h, 10 uM	270	103	186
PHH, 24 h, 10 uM	107	44	64
SKB, 24 h, 10 uM	109	47	70
VCAP, 6 h, 10 uM	218	78	149
All cell lines	2839	1102	1838

For each gene expression dataset both Random Forest (RF) and Support Vector Machine (SVM) classification models were developed. As for models generated for other descriptors, we used those labelled as vMostConcern for the positive class and those labelled as vNoConcern for the negative class, with the resulting dataset termed "DILIrank (-vLessConcern)". The model development workflow was the same as that used for other descriptors (**Methods, Model Generation**), except that replicates were kept together during both the outer 10-fold stratified splits (StratifiedKFold in sklearn) and the inner 5-fold cross-

validation splits (GroupKFold in sklearn). The LOCO-CV scheme based on Tanimoto similarity employed previously was not used.

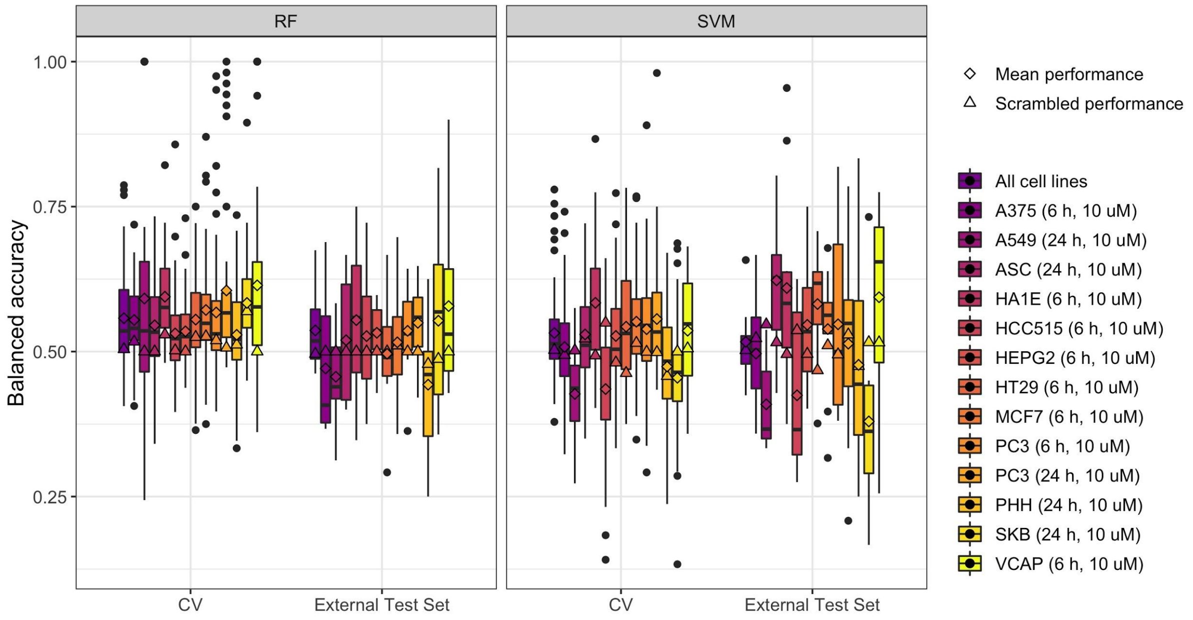

Figure SI_GEX 1: DILI label prediction performance (balanced accuracy) of RF and SVM models trained using different gene expression datasets. Models were trained using the vMostConcern and vNoConcern classes described in Table SI_GEX 1. Performance is stable between the 5-fold CV and external test set. However, the prediction accuracies were similar to that achieved by y-scrambling models demonstrating that the models did not perform much better than expected at random.

It can be seen from Figure SI_GEX 1 that across all datasets the RF and SVM models did not achieve meaningfully higher prediction balanced accuracies than y-scrambling models with a median balanced accuracy (across the median balanced accuracy per dataset) of only 0.53 (CV) and 0.52 (External Test Set). RF models trained using the SKB cell line (24 h, 10 uM) achieved the best CV performance (0.59 median balanced accuracy), whilst SVM models trained using the VCAP cell line (6 h, 10 uM) achieved the best external test set performance (0.66 median balanced accuracy).

Across the gene expression datasets, the balanced accuracies observed are far lower than those observed using descriptors derived from chemical structure. However, we stress that this observation is the result of a particular set of input descriptors, combined with the data processing and machine learning model generation described here. Additionally, it should be noted that there is evidence that machine learning models using descriptors derived from gene expression data as input can be predictive of DILI (1).

1. Kohonen P, Parkkinen JA, Willighagen EL, Ceder R, Wennerberg K, Kaski S, et al. A transcriptomics data-driven gene space accurately predicts liver cytopathology and drug-induced liver injury. Nat Commun. 2017 Jul 3;8:15932.
