# Supplementary figures and images for: Prediction and mechanistic analysis of drug-induced liver injury (DILI) based on chemical structure
Source: Biol Direct. 2021 Jan 18;16:6. doi: 10.1186/s13062-020-00285-0 (PMC7814730; doi:10.1186/s13062-020-00285-0)

Balanced Accuracy

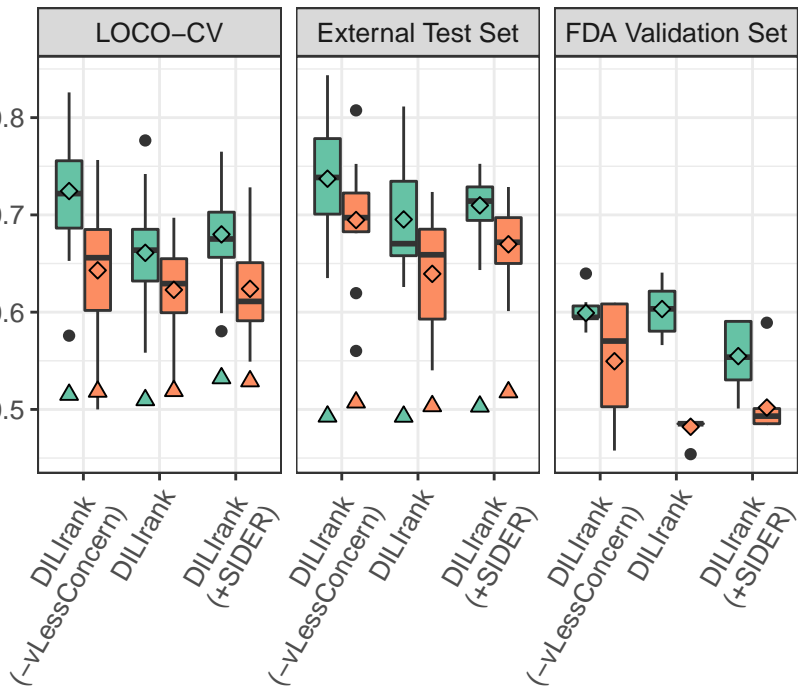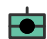

RF

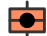

SVM

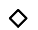

Mean performance

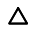

Scrambled performance

Supplement: Supplementary file 2 — Additional file 2: Figure S1. DILI label prediction performance (balanced accuracy) of RF and SVM models trained using the DILIrank (−vLessConcern) dataset and Mordred molecular descriptors for 5-fold LOCO-CV, external test set, and FDA validation set (Methods). The balanced accuracy for 5-fold internal cross-validation, external test set, and FDA validation set for 10 models trained using different training data sets (DILIrank (−vLessConcern), DILIrank, DILIrank (+SIDER)) and training dataset splits is shown via whisker plots. The median model performance of 3 y-scrambled models is shown as triangles for cross-validation and external test set. Predictive accuracy is stable between cross-validation and external test set, but a distinct drop in predictive accuracy is observed when predicting the FDA validation set. [file 13062_2020_285_MOESM2_ESM.pdf]

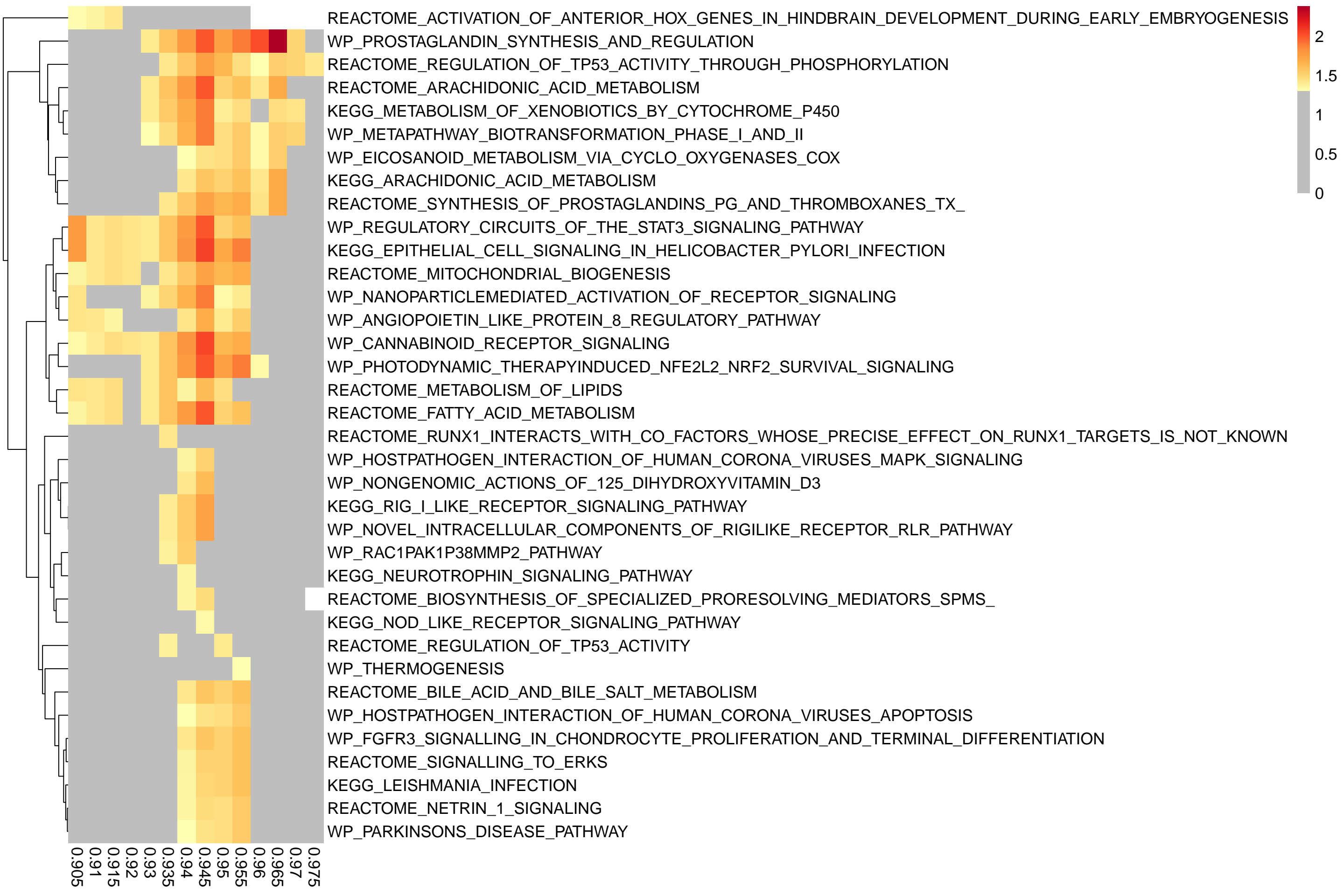

Supplement: Supplementary file 4 — Additional file 4: Figure S3. Enriched pathways across different feature importance cutoffs for RF using the DILIrank (−vLessConcern) dataset. Enriched pathways are shown across different feature importance cutoffs which are identified by the percentile of DILI-enriched protein targets covered. Significant pathways (FDR < 0.05) are colored by -log (FDR), pathways without any gene present are shown in white and insignificant ones in grey. Regulation of TP53 through phosphorylation is the pathway conserved at the highest threshold identifying significant pathways. Other identified pathways include arachidonic acid metabolism and prostaglandin synthesis. [file 13062_2020_285_MOESM4_ESM.pdf]

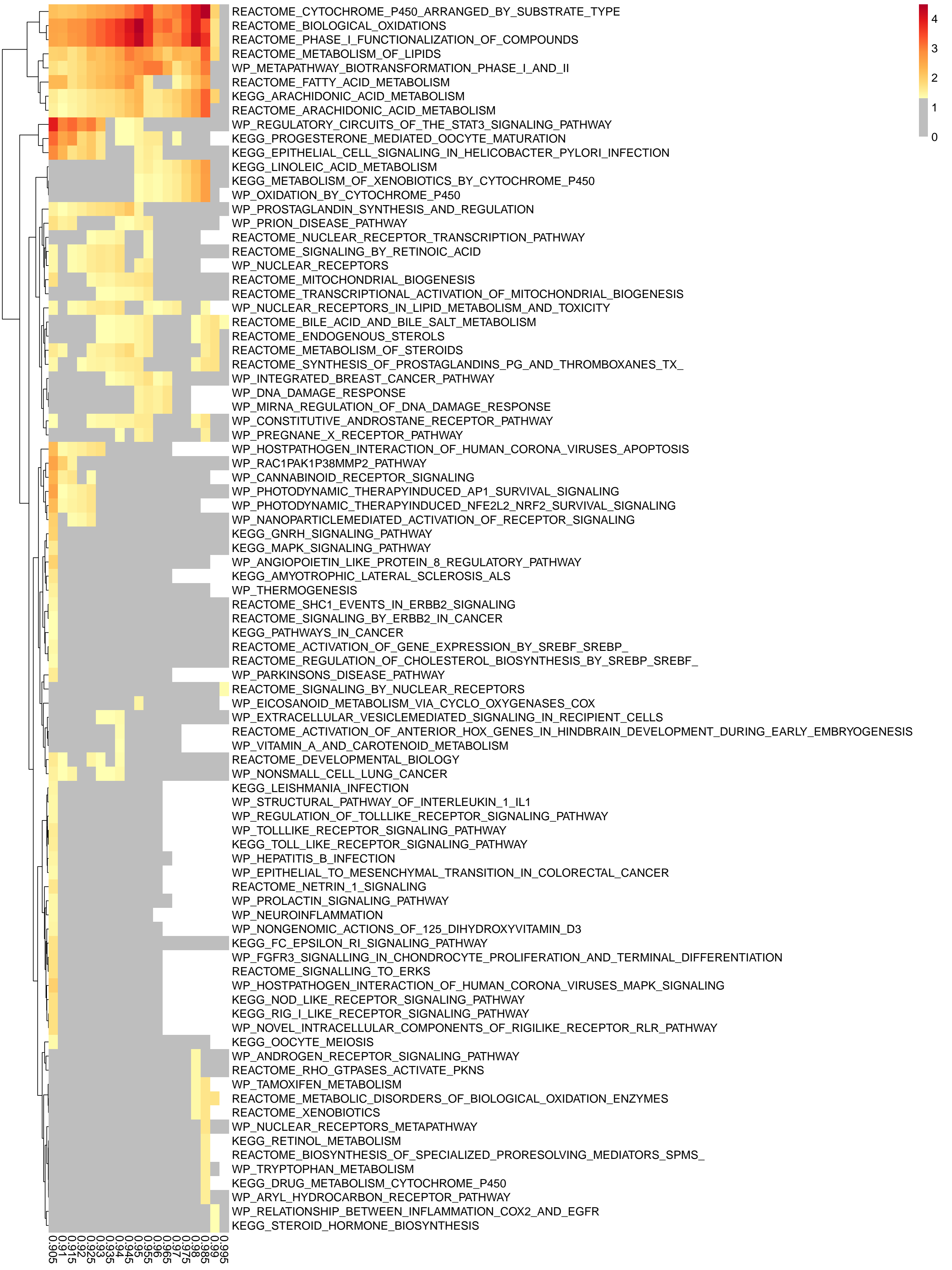

Supplement: Supplementary file 5 — Additional file 5: Figure S4. Enriched pathways across different feature importance cutoffs for SVM using the DILIrank (−vLessConcern) dataset. Enriched pathways are shown across different feature importance cutoffs which are identified by the percentile of DILI-enriched protein targets covered. Significant pathways (FDR < 0.05) are colored by -log (FDR), pathways without any gene present are shown in white and insignificant ones in grey. While some pathways are only significant at high thresholds, such as steroid hormone biosynthesis, others are only found at lower thresholds, e.g. TLR signaling. Additionally, a set of pathways including biotransformation, cytochrome 450 and arachidonic acid metabolism are observed across the majority of thresholds. [file 13062_2020_285_MOESM5_ESM.pdf]
