# Supplementary material for: Prediction and mechanistic analysis of drug-induced liver injury (DILI) based on chemical structure
Source: Biol Direct. 2021 Jan 18;16:6. doi: 10.1186/s13062-020-00285-0 (PMC7814730; doi:10.1186/s13062-020-00285-0)
Supplement: Supplementary file 6 — Additional file 6: Table S1. Performance of models trained using the DILIrank (−vLessConcern) dataset. Shown is mean ± standard deviation for 7 metrics (MCC - Matthew’s Correlation Coefficient, PRAUC - Precision-Recall Area Under Curve, ROCAUC - Receiver-Operator-Characteristic Area Under Curve) for models trained using ECFP4, Mordred molecular descriptors (MD), and protein target descriptors (PT). Row names correspond to the descriptor type, algorithm, and the test set - external test set (ET), FDA validation set (FDA). The best external test set and FDA validation set performance per metric are shown in bold. For the FDA validation set, PRAUC and ROCAUC were not available as only the confusion matrices of the predictions were provided by CAMDA. The model trained using SVM and ECFP4 descriptors achieved the best performance over the FDA validation set. [file 13062_2020_285_MOESM6_ESM.pdf]

	Accuracy	Balanced accuracy	Recall	Precision	MCC	PRAUC	ROCAUC
ECFP4 RF ET	0.753±0.034	0.746±0.032	0.689±0.065	0.736±0.070	0.500±0.070	**0.822±0.059**	**0.847±0.038**
ECFP4 RF FDA	0.600±0.022	0.561±0.017	0.435±0.041	0.427±0.026	0.123±0.035	-	-
ECFP4 SVM ET	**0.763±0.026**	**0.759±0.027**	**0.724±0.084**	0.736±0.056	**0.523±0.054**	0.778±0.065	0.824±0.034
ECFP4 SVM FDA	**0.673±0.000**	**0.667±0.000**	0.647±0.000	**0.524±0.000**	**0.322±0.000**	-	-
MD RF ET	0.748±0.059	0.737±0.063	0.655±0.118	0.740±0.073	0.487±0.122	0.770±0.084	0.813±0.052
MD RF FDA	0.637±0.019	0.599±0.018	0.476±0.019	0.477±0.026	0.199±0.036	-	-
MD SVM ET	0.701±0.069	0.695±0.068	0.649±0.082	0.662±0.093	0.393±0.140	0.644±0.110	0.718±0.064
MD SVM FDA	0.576±0.063	0.550±0.066	0.465±0.076	0.405±0.076	0.097±0.128	-	-
PT RF ET	0.750±0.049	0.741±0.052	0.671±0.095	0.732±0.059	0.489±0.101	0.787±0.080	0.821±0.043
PT RF FDA	0.633±0.029	0.589±0.035	0.447±0.074	0.469±0.045	0.181±0.069	-	-
PT-SVM ET	0.757±0.042	0.753±0.039	0.722±0.076	0.727±0.073	0.511±0.081	0.815±0.062	0.837±0.043
PT SVM FDA	0.547±0.016	0.576±0.03	**0.671±0.074**	0.406±0.02	0.147±0.059	-	-
