# Supplementary material for: Prediction and mechanistic analysis of drug-induced liver injury (DILI) based on chemical structure
Source: Biol Direct. 2021 Jan 18;16:6. doi: 10.1186/s13062-020-00285-0 (PMC7814730; doi:10.1186/s13062-020-00285-0)
Supplement: Supplementary file 7 — Additional file 7: TableS2. Proteins with high feature importance in RF and SVM, and links to DILI. The 19 proteins with the highest feature importance in RF or SVM models are shown. The feature importance is shown in bold if the protein ranked among the top 19 in the respective model. Many proteins identified possess known functions in liver drug metabolism and cell stress. Those proteins with plausible involvement in DILI are indicated in italics. [file 13062_2020_285_MOESM7_ESM.pdf]

Symbol	Name	Protein Classification	Feature Importance		Known/plausible in DILI?	Citation(s)
			RF	SVM		
ADORA1	Adenosine A1 receptor	GPCR	**0.00384**	-0.47612	Involvement in protection against DILI for ADORA2 - Putative novel target?	Chiang, D.J. et al. (2013)[1]
AKR1B1	Aldose reductase	Oxidoreductases	**0.00447**	0.28545	Oxidative stress and apoptosis	Ahmed, M.M. et al. (2016)[2]
AKR1C2	Aldo-keto reductase family 1 member C2	Oxidoreductases	**0.00244**	-0.01159	Drug metabolism, Phase II Reaction	Barski, O.A. et al. (2008)[3]; Chen, W.D. et al. (2012)[4]
AKR1C3	Aldo-keto-reductase family 1 member C3	Oxidoreductases	**0.00441**	**0.48452**	Drug metabolism, Phase II Reaction	Barski, O.A. et al. (2008)[3]; Chen, W.D. et al. (2012)[4]
APBA1	Voltage-gated N-type calcium channel alpha-1B subunit/Amyloid beta A4 precursor protein-binding family A member 1	Transporter	**0.00542**	0.04074	No Involvment in DILI	
AR	Androgen Receptor	NHR	0.00063	**0.39500**	Sex discrepancy in DILI	Sutti, S. et al. (2018)[5]
C1R	Complement C1r	Protease	**0.00548**	0.02562	No Involvment in DILI	
CCNB2	Cyclin-dependent kinase 1/cyclin B2	Other	0.00097	**0.35835**	Overexpressed during liver regeneration	Lu, X.P. et al.(1992)[6]
CCNB3	Cyclin-dependent kinase 1/cyclin B3	Other	0.00076	**0.35835**	No Involvment in DILI	
CDC25A	Dual specificity phosphatase Cdc25A	Phosphatase	0.00067	**0.32319**	Overexpressed in hepatocellular carcinoma cells	Xundi, X. et al. (2003)[7]
CLK1	Dual specificty protein kinase CLK1	Kinase	**0.00841**	0.08904	No Involvment in DILI	
CSNK2A2	Casein kinase II alpha (prime)	Kinase	**0.00285**	0.01077	No Involvment in DILI	
CXCR2	Interleukin-8 receptor B	GPCR	0.00209	**0.38108**	Activation in patients with chronic liver diseases	Zimmermann, H.W. et al. (2011)[8]
CYP19A1	Cytochrome P450 19A1	Oxidoreductases	0.00128	**0.45375**	No Involvment in DILI	
CYP1A2	Cytochrome P450 1A2	Oxidoreductases	0.00193	**0.43226**	Drug metabolism, high expression in liver	Zanger, U.M. et al. (2013)[9]
CYP2C9	Cytochrome P450 2C9	Oxidoreductases	**0.00241**	**0.42051**	Drug metabolism, high expression in liver	Zanger, U.M. et al. (2013)[9]
DYRK2	Dual-specificity tyrosine-phosphorylation regulated kinase 2	Kinase	**0.00265**	0.21174	No Involvment in DILI	
EGFR	Epidermal growth factor receptor erbB1	Kinase	0.00147	**0.55702**	No Involvment in DILI	

EIF2AK4	Eukaryotic translation initiation factor 2-alpha kinase 4	Kinase	**0.00248**	0.04233	Regulation of acute and chronic liver injury	Arriazu, E. et al. (2013)[10]
ESRRA	Estrogen-related receptor alpha	NHR	0.00091	**0.34558**	Loss of activity promotes hepatocellular carcinoma	Eui-Ju Hong, E.-J. et al. (2013)[11]
FABP4	Fatty acid binding protein adipocyte	Transporter	0.00063	**0.30870**	Not usually expressed in liver, expressed by hepatocellular carcinoma cells	Thompson,K. J. et al. (2018)[12]
FBP1	Fructose-1	6-bisphosphatas e	**0.00246**	-0.12770	No Involvment in DILI	
FGR	Tyrosine-protein kinase FGR	Kinase	**0.00291**	0.13935	No Involvment in DILI	
MAPK11	MAP kinase p38 beta	Kinase	**0.00593**	0.28874	Cellular stress response (Autophagy/Oxidative stress)	Yang, X. et al. (2015)[13]; Huang, Y. et al. (2019)[14]
MPL	Thrombopoietin receptor	Other	**0.00392**	0.23564	No Involvment in DILI	
NCOA2	Peroxisome proliferator-activated receptor gamma/Nuclear receptor coactivator 2	Other	0.00079	**0.48584**	No Involvment in DILI	
NQO2	Quinone reductase 2	Oxidoreductases	**0.00300**	**0.38398**	Oxidative stress	Miettinen, T.P. et al. (2014)[15]
P4HTM	Hypoxia-inducible factor prolyl 4-hydroxylase	Other	**0.00286**	0.01138	Inactivation has a protective role for alcohol induced liver damages	Laitakari, A. et al. (2019)[16]
PLA2G1B	Phospholipase A2 group 1B	Hydrolases	0.00148	**0.67637**	Protection against Fas-induced iver injuries	Li, G. et al. (2011)[17]
PRKAB1	AMP-activated protein kinase	beta-1 subunit	**0.00340**	0.19311	Protective role in liver rigeneration	Huang, J. et al. (2018)[18]
PRNP	Prion protein	Other	0.00082	**0.43342**	No Involvment in DILI	
PTGES	Prostaglandin E synthase	Isomerases	**0.00330**	0.15025	Inhibitors cause severe DILI	Jin, Y. et al. (2018)[19]; Norman, et al. B.H. (2018)[20]
RARG	Retinoic acid receptor gamma	NHR	0.00153	**0.32604**	Higher expression in nonalcoholic steatohepatitis	Erin E. E. et al. (2018)[21]
RPS6KA 6	Ribosomal protein S6 kinase alpha 6	Kinase	0.00053	**0.41260**	Prevention and treatment of liver injuries and fibrosis	Buck, M. et al. (2008)[22]
TBXAS1	Thromboxane-A synthase	Isomerases	0.00071	**0.45820**	Production of hepatic injury during hepatic stress	Yokoama, Y. et al. (2005)[23]

1. Chiang, D. J. *et al.* Adenosine 2A Receptor Antagonist Prevented and Reversed Liver Fibrosis in a Mouse Model of Ethanol-Exacerbated Liver Fibrosis. *PLoS ONE* **8**, e69114 (2013).
2. Ahmed, M. M., Al-Obosi, J., Osman, H. & Shayoub, M. Overexpression of Aldose Reductase Render Mouse Hepatocytes More Sensitive to Acetaminophen Induced Oxidative Stress and Cell Death. *Indian J. Clin. Biochem.* **31**, 162–170 (2016).
3. Barski, O. A., Tipparaju, S. M. & Bhatnagar, A. The Aldo-Keto Reductase Superfamily and its Role in Drug Metabolism and Detoxification. *Drug Metab. Rev.* **40**, 553–624 (2008).
4. Chen, W.-D. & Zhang, Y. Regulation of aldo–keto reductases in human diseases. *Front. Pharmacol.* **3**, 35 (2012).
5. Sutti, S. & Tacke, F. Liver inflammation and regeneration in drug-induced liver injury: sex matters! *Clin. Sci.* **132**, 609–613 (2018).
6. Lu, X. *et al.* Induction of cyclin mRNA and cyclin-associated histone H1 kinase during liver regeneration. *J. Biol. Chem.* **267**, 2841–2844 (1992).
7. Xu, X. *et al.* Overexpression of CDC25A phosphatase is associated with hypergrowth activity and poor prognosis of human hepatocellular carcinomas. *Clin. Cancer Res.* **9**, 1764–1772 (2003).
8. Zimmermann, H. W. *et al.* Interleukin-8 Is Activated in Patients with Chronic Liver Diseases and Associated with Hepatic Macrophage Accumulation in Human Liver Fibrosis. *PLoS ONE* **6**, e21381 (2011).
9. Zanger, U. M. & Schwab, M. Cytochrome P450 enzymes in drug metabolism: regulation of gene expression, enzyme activities, and impact of genetic variation. *Pharmacol. Ther.* **138**, 103–141 (2013).
10. Arriazu, E. *et al.* GCN2 kinase is a key regulator of fibrogenesis and acute and chronic liver injury induced by carbon tetrachloride in mice. *Lab. Invest.* **93**, 303–310 (2013).
11. Hong, E.-J., Levasseur, M.-P., Dufour, C. R., Perry, M.-C. & Giguère, V. Loss of estrogen-related receptor α promotes hepatocarcinogenesis development via metabolic and inflammatory disturbances. *Proc. Natl. Acad. Sci.* **110**, 17975–17980 (2013).
12. Thompson, K. J. *et al.* Altered fatty acid-binding protein 4 (FABP 4) expression and function in human and animal models of hepatocellular carcinoma. *Liver Int.* **38**, 1074–1083 (2018).
13. Yang, X. *et al.* Autophagy protects against dasatinib-induced hepatotoxicity via p38 signaling.

Oncotarget **6**, 6203 (2015).

14. Huang, Y. *et al.* The Increase of ROS Caused by the Interference of DEHP with JNK/p38/p53 Pathway as the Reason for Hepatotoxicity. *Int. J. Environ. Res. Public. Health* **16**, 356 (2019).
15. Miettinen, T. P. & Björklund, M. NQO2 Is a Reactive Oxygen Species Generating Off-Target for Acetaminophen. *Mol. Pharm.* **11**, 4395–4404 (2014).
16. Laitakari, A. *et al.* Systemic inactivation of hypoxia-inducible factor prolyl 4-hydroxylase 2 in mice protects from alcohol-induced fatty liver disease. *Redox Biol.* **22**, 101145 (2019).
17. Li, G., Chen, W., Han, C. & Wu, T. Cytosolic phospholipase A2α protects against Fas-but not LPS-induced liver injury. *J. Hepatol.* **55**, 1281–1290 (2011).
18. Huang, J. *et al.* Potential roles of AMP-activated protein kinase in liver regeneration in mice with acute liver injury. *Mol. Med. Rep.* (2018) doi:10.3892/mmr.2018.8522.
19. Jin, Y. *et al.* Dose-dependent acute liver injury with hypersensitivity features in humans due to a novel microsomal prostaglandin E synthase 1 inhibitor. *Br. J. Clin. Pharmacol.* **84**, 179–188 (2018).
20. Norman, B. H. *et al.* Identification and Mitigation of Reactive Metabolites of 2-Aminoimidazole-Containing Microsomal Prostaglandin E Synthase-1 Inhibitors Terminated Due to Clinical Drug-Induced Liver Injury. *J. Med. Chem.* **61**, 2041–2051 (2018).
21. Elbel, E. E. *et al.* Hepatic nuclear receptor expression associates with features of histology in pediatric nonalcoholic fatty liver disease. *Hepatol. Commun.* **2**, 1213–1226 (2018).
22. Buck, M. Targeting ribosomal S-6 kinase for the prevention and treatment of liver injury and liver fibrosis. *Drug News Perspect.* **21**, 301–306 (2008).
23. Yokoyama, Y., Nimura, Y., Nagino, M., Bland, K. I. & Chaudry, I. H. Role of thromboxane in producing hepatic injury during hepatic stress. *Arch. Surg.* **140**, 801–807 (2005).
