# Supplementary material for: Prediction and mechanistic analysis of drug-induced liver injury (DILI) based on chemical structure
Source: Biol Direct. 2021 Jan 18;16:6. doi: 10.1186/s13062-020-00285-0 (PMC7814730; doi:10.1186/s13062-020-00285-0)
Supplement: Supplementary file 8 — Additional file 8: TableS3. Pathways with high feature importance in RF and SVM, and links to DILI. The overrepresented gene sets for 19 proteins with the highest feature importance in RF or SVM models are shown. Many pathways identified possess known functions in liver drug metabolism and cell stress. [file 13062_2020_285_MOESM8_ESM.pdf]

Pathway	Description	RF		SVM		Known/plausible in DILI?	Citation(s)
		Padj	Gene Ratio	Padj	Gene Ratio		
Arachidonic acid metabolism	Kegg_arachidonic_acid_metabolism	-	-	0.0111	4/19	Involvement in Acetaminophen and galactosamine/endotoxin induced liver injuries	Suciu, M. et al. 2015 (2015)[1]; Xue-Jun M. et al. (1994)[2]
	Reactome_arachidonic_acid_metabolism	-	-	0.0124	4/19		
Biotransformation	Wp_metapathway_biotransformation_phase_i_and_ii	0.0318	4/17	0.02	4/19	Biotransformation reactions on drugs responsible for liver injuries	Xinsheng, G. et al. (2012)[3]; Mitchell, J.R. et al. (1976)[4]; Yuan, L. et al (2013)[5]
	Reactome_phase_i_functionalization_of_compounds	-	-	0.0010	6/19		
	Reactome_biological_oxidations	-	-	0.0014	6/19		
Cytochrome p450	Kegg_metabolism_of_xenobiotics_by_cytochrome_p450	0.0374	3/17	0.0272	3/19	Involvment in drugs metabolism and pathogenesis of several liver diseases. Inhibition of CYP is an indicator of drug-induced hepatotoxicity	Villeneuve, J.P. et al. (2004)[6]; Feng, S. et al. (2013)[7]
	Reactome_cytochrome_p450_arranged_by_substrate_type	-	-	0.0010	5/19		
	Wp_oxidation_by_cytochrome_p450	-	-	0.0272	3/19		
Fatty acid metabolism	Reactome_fatty_acid_metabolism	-	-	0.0374	4/19	Involvment in Acetaminophen and Divalproex sodium induced liver injuries	Suciu, M. et al. (2015)[1]; Wang W. et al. (2012)[8]
Linoleic acid metabolism	Kegg_linoleic_acid_metabolism	-	-	0.0237	3/19	Conjugated Linoleic Acid provokes fulminant hepatitis	Bilal, M. et al. (2015)[9]; Nortadas, R. et al. (2012)[10]
Metabolism of lipids	Reactome_metabolism_of_lipids	-	-	0.0047	9/19	Involvement in drug-induced toxicity	Xu, S. et al. (2019)[11]; Begriche K. et al. (2011)[12]
Nuclear receptors in lipid metabolism and toxicity	Wp_nuclear_receptors_in_lipid_metabolism_and_toxicity	-	-	0.0292	3/19	Nuclear receptors control all phases of hepatic drug metabolism and are xenobiotic sensors	Rudraiah, S. et al. (2016)[13]
Progesterone mediated oocyte maturation	Kegg_progesterone_mediated_oocyte_maturation	-	-	0.04381	4/19	Progesterone contributes to worsen DILIs	Toyoda, Y. et al. (2012)[14]

Prostaglandin synthesis and regulation	Wp_prostaglandin_synthesis_and_regulation	0.0318	4/17	-	-	Role in liver injuries	Peltekian,K.(1996)[15] et al; Cavar, I. et al. (2010)[16]
Regulation of tp53 activity through phosphorylation	Reactome_regulation_of_tp53_activity_through_phosphorylation	0.0318	4/17	-	-	Involvement in Acetaminophen induced liver injuries	Huo Y. et al. (2017)[17]

1. Suciu, M. *et al.* Acetaminophen-induced liver injury: Implications for temporal homeostasis of lipid metabolism and eicosanoid signaling pathway. *Chem. Biol. Interact.* **242**, 335–344 (2015).
2. Xue-jun, M. & Jia-long, W. Arachidonic acid metabolism in galactosamine/endotoxin induced acute liver injury in rats. *J. Tongji Med. Univ.* **14**, 169–172 (1994).
3. Gu, X. & Manautou, J. E. Molecular mechanisms underlying chemical liver injury. *Expert Rev. Mol. Med.* **14**, (2012).
4. Mitchell, J. R., Snodgrass, W. R. & Gillette, J. R. The role of biotransformation in chemical-induced liver injury. *Environ. Health Perspect.* **15**, 27–38 (1976).
5. Yuan, L. & Kaplowitz, N. Mechanisms of drug-induced liver injury. *Clin. Liver Dis.* **17**, 507–518, vii (2013).
6. Pichette, J.-P. V. and V. Cytochrome P450 and Liver Diseases. *Current Drug Metabolism* vol. 5 273–282 https://www.eurekaselect.com/61864/article (2004).
7. Feng, S. & He, X. Mechanism-based inhibition of CYP450: an indicator of drug-induced hepatotoxicity. *Curr. Drug Metab.* **14**, 921–945 (2013).
8. Wang, W. *et al.* Involvement of fatty acid metabolism in the hepatotoxicity induced by divalproex sodium: *Hum. Exp. Toxicol.* (2012) doi:10.1177/0960327112444477.
9. Bilal, M., Patel, Y., Burkitt, M. & Babich, M. Linoleic Acid Induced Acute Hepatitis: A Case Report and Review of the Literature. *Case Reports in Hepatology* vol. 2015 e807354 https://www.hindawi.com/journals/crihep/2015/807354/ (2015).
10. Nortadas, R. & Barata, J. Fulminant hepatitis during self-medication with conjugated linoleic acid. *Ann. Hepatol.* **11**, 265–267 (2012).

11. Xu, S. *et al.* Lipidomic Profiling Reveals Disruption of Lipid Metabolism in Valproic Acid-Induced Hepatotoxicity. *Front. Pharmacol.* **10**, (2019).
12. Begriche, K., Massart, J., Robin, M.-A., Borgne-Sanchez, A. & Fromenty, B. Drug-induced toxicity on mitochondria and lipid metabolism: mechanistic diversity and deleterious consequences for the liver. *J. Hepatol.* **54**, 773–794 (2011).
13. Rudraiah, S., Zhang, X. & Wang, L. Nuclear Receptors as Therapeutic Targets in Liver Disease: Are We There Yet? *Annu. Rev. Pharmacol. Toxicol.* **56**, 605–626 (2016).
14. Toyoda, Y. *et al.* Mechanism of Exacerbative Effect of Progesterone on Drug-Induced Liver Injury. *Toxicol. Sci.* **126**, 16–27 (2012).
15. Peltekian, K. M., Makowka, L., Williams, R., Blendis, L. M. & Levy, G. A. Prostaglandins in liver failure and transplantation: Regeneration, immunomodulation, and cytoprotection. *Liver Transpl. Surg.* **2**, 171–184 (1996).
16. Cavar, I., Kelava, T., Vukojevic, K., Saraga-Babic, M. & Čulo, F. The role of prostaglandin E2 in acute acetaminophen hepatotoxicity in mice. *Histol. Histopathol.* **25**, 819–30 (2010).
17. Huo, Y. *et al.* Protective role of p53 in acetaminophen hepatotoxicity. *Free Radic. Biol. Med.* **106**, 111–117 (2017).
