# Supplementary material for: Prediction and mechanistic analysis of drug-induced liver injury (DILI) based on chemical structure
Source: Biol Direct. 2021 Jan 18;16:6. doi: 10.1186/s13062-020-00285-0 (PMC7814730; doi:10.1186/s13062-020-00285-0)
Supplement: Supplementary file 9 — Additional file 9: TableS4. Top significant structural alerts (p-value ≤0.05). The following quality metrics are shown: precision, coverage in DILI positive compounds (%), and number of Drugbank [44] approved compounds with the substructure are shown. *MoSS substructure notation is in the form of subgraphs with only heavy atoms, which are neither SMILES nor SMARTS. [file 13062_2020_285_MOESM9_ESM.pdf]

Structure	SMARTS (*)	Precision	Source	p-value	Coverage in DILI compounds %	Number Drugbank Approved Drugs
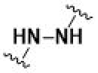	NN	1	Liu et al.	0.00001	7.47	37
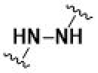	N-N	1	MoSS	0.00001	7.47	37
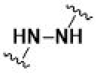	NN	1	SARpy	0.00001	7.47	37
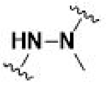	N(-N)-C	1	MoSS	0.00009	6.32	33
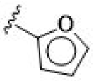	c1ccco1	1	SARpy	0.00020	5.75	19
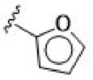	o1:c(:c:c:c:1)-C	1	MoSS	0.00048	5.17	17
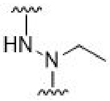	N(-N)-C-C	1	MoSS	0.00115	4.60	26
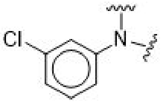	Cl-c1:c:c(:c:c:c:1)-N	1	MoSS	0.00270	4.02	35
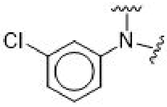	Nc4cccc(Cl)c4	1	SARpy	0.00270	4.02	35
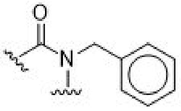	N(-C(-C)=O)-C-c1:c:c:c:c:c:1	1	MoSS	0.00635	3.45	28
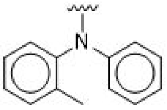	N(-c1:c(:c:c:c:c:1)-C)-c1:c:c:c:c:c:1	1	MoSS	0.00635	3.45	20
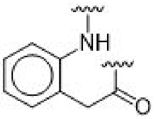	Nc1c(CC(=O))cccc1	1	SARpy	0.00635	3.45	11
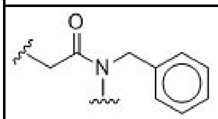	O=C(CC)NCc1ccccc1	1	SARpy	0.00635	3.45	22
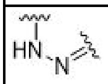	N(-N)=C	1	MoSS	0.01488	2.87	12
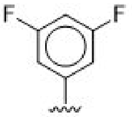	F-c1:c:c(:c:c:c:1)-F	1	MoSS	0.01488	2.87	16

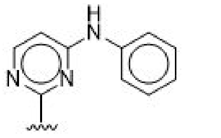	n1:c:n:c:c:c:1-N-c1:c:c:c:c:c:1	1	MoSS	0.01488	2.87	10
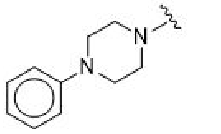	N1(-C-C-N-C-C-1)-c1:c:c:c:c:c:1	1	MoSS	0.01488	2.87	28
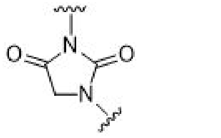	N1-C(-N-C-C-1=O)=O	1	MoSS	0.01488	2.87	11
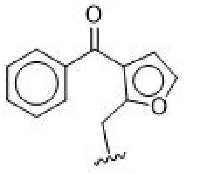	o1:c(:c(:c:c:1)-C(-c1:c:c:c:c:c:1)=O)-C-C	1	MoSS	0.01488	2.87	3
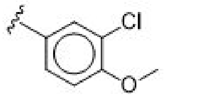	Cl-c1:c(:c:c:c:c:1)-O-C	1	MoSS	0.03476	2.30	9
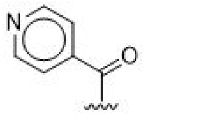	n1:c:c:c(:c:c:1)-C=O	1	MoSS	0.03476	2.30	5
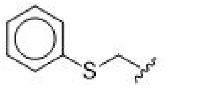	S(-c1:c:c:c:c:c:1)-C-C	1	MoSS	0.03476	2.30	15
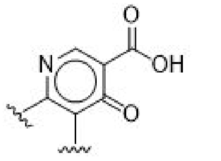	n1:c:c(:c(:c:c:1)=O)-C(-O)=O	1	MoSS	0.03476	2.30	21
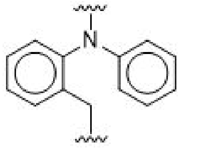	N(-c1:c(:c:c:c:c:1)-C-C)-c1:c:c:c:c:c:1	1	MoSS	0.03476	2.30	11
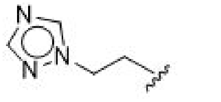	n1(:n:c:n:c:1)-C-C-C	1	MoSS	0.03476	2.30	10
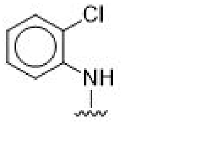	Cl-c1:c(:c:c:c:c:1)-N	1	MoSS	0.03476	2.30	21
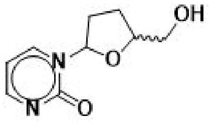	n1(:c(:n:c:c:c:1)=O)-C1-O-C(-C-C-1)-C-O	1	MoSS	0.03476	2.30	13
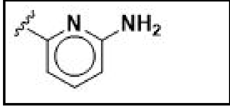	n1:c(:c:c:c:c:1)-N	1	MoSS	0.03476	2.30	41

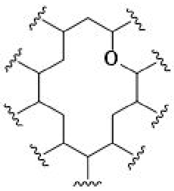	O1-C-C-C-C-C-C(-C-C-C-C-C-C-C-1)-C	1	MoSS	0.03476	2.30	9
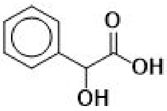	a[C!R]C(=O)[OH]	0.9167	Liu et al.	0.00063	6.32	27
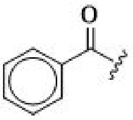	a[C!R](=O)a	0.9167	Liu et al.	0.00063	6.32	25
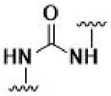	NC(=O)N	0.9167	SARpy	0.00063	6.32	80
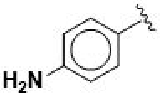	c1ccccc1[NH2]	0.8571	Liu et al.	0.02825	3.45	62
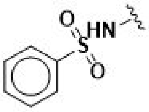	NS(=O)(=O)c2ccccc2	0.8182	SARpy	0.01029	5.17	76
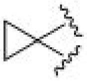	C1CC1	0.7777	SARpy	0.03866	4.02	60
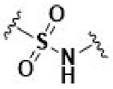	[#6]S(=O)(=O)N[#6]	0.7059	Liu et al.	0.01974	6.90	89
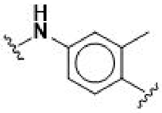	Cc1cccc(N)c1	0.6875	SARpy	0.03384	6.32	95
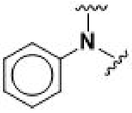	c1ccc(N)cc1	0.6471	SARpy	0.00001	31.61	422
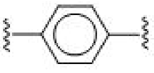	c1ccccc1	0.4737	SARpy	0.00771	77.59	1400
